# Estimating the combining ability and genetic parameters for growth habit, yield, and fiber quality traits in some Egyptian cotton crosses

**DOI:** 10.1186/s12870-023-04131-z

**Published:** 2023-03-02

**Authors:** M.S. Abdel-Aty, F. A. Sorour, W. M. B. Yehia, H. M. K. Kotb, Ahmed M. Abdelghany, Sobhi F. Lamlom, Adnan Noor Shah, Nader R. Abdelsalam

**Affiliations:** 1 Agronomy Department, Faculty of Agriculture, Kafr El-Sheikh University, Kafr El-Sheikh, 33516 Egypt; 2 Cotton Breeding Department, Cotton Research Institute, Agriculture Research Center, Giza, Egypt; 3 Crop Science Department, Faculty of Agriculture, Damanhour University, Damanhour, 22516 Egypt; 4 Plant Production Department, Faculty of Agriculture (Saba Basha), Alexandria University, Alexandria, 21531 Egypt; 5 Department of Agricultural Engineering, Khwaja Fareed University of Engineering and Information Technology, Rahim Yar Khan, Punjab, 64200 Pakistan; 6 Agricultural Botany Department, Faculty of Agriculture (Saba Basha), Alexandria University, Alexandria, 21531 Egypt

**Keywords:** Hybrid, Genotypes, Mating, Genetic diversity, Cotton, Fiber quality, Fiber length

## Abstract

It is crucial to understand how targeted traits in a hybrid breeding program are influenced by gene activity and combining ability. During the three growing seasons of 2015, 2016, and 2017, a field study was conducted with twelve cotton genotypes, comprised of four testers and eight lines. Thirty-two F1 crosses were produced in the 2015 breeding season using the line x tester mating design. The twelve genotypes and their thirty-two F1 crosses were then evaluated in 2016 and 2017. The results demonstrated highly significant differences among cotton genotypes for all the studied traits, showing a wide range of genetic diversity in the parent genotypes. Additionally, the line-x-tester interaction was highly significant for all traits, suggesting the impact of both additive and non-additive variations in gene expression. Furthermore, the thirty-two cotton crosses showed high seed cotton output, lint cotton yield, and fiber quality, such as fiber length values exceeding 31 mm and a fiber strength above 10 g/tex. Accordingly, selecting lines and testers with high GCA effects and crosses with high SCA effects would be an effective approach to improve the desired traits in cotton and develop new varieties with excellent yield and fiber quality.

## Introduction

Cotton is the world's most significant fiber commodity and Egypt's most important cash crop. In 2022, the global total area harvested, yield, and production of cotton were 32.65 million hectares, 811 kg per hectare, and 121.57 million 480-pound bales, respectively. In Egypt, the overall harvested area, yield, and production of cotton were 0.1 million hectares, 718 kg per hectare, and 0.33 million 480-pound bales. Cotton production fell 8.83% globally and 53.49% in Egypt in the 2021/22 cropping season compared to the previous year (USDA, 2022). The major aim of cotton breeders is to look for and select genotypes with high-yield traits, as well as utilizing the genetic diversity that Egyptian cultivars still have untapped. Significant effort has been made in Egypt to improve cotton's productivity and quality attributes [1]. Moreover, using the genetic components of any breeding germplasm and genetic resources recorded complete and useful information for cotton breeders to choose the suitable breeding procedure for developing improved crosses [2–4].

To create a breeding program for the economic exploitation of heterosis for hybrid crop development or variety creation, Parents need to be genetically superior, physiologically effective, and have improved general and particular combining abilities [5]. Heterosis is a phenomenon when hybrid offspring perform better than their parental inbred lines [6]. It substantially contributes to the specific combining ability (SCA) variance, which is a measure of dominance variance and the existence of a substantial degree of dominance variance has been reported [7]. This is essential for undertaking a heterosis breeding program [8]. Choosing appropriate parental genotypes is critical for allocating genetic resources to the most promising varieties and increasing the efficiency of breeding efforts. Line x tester analysis has been widely utilized by plant breeders to evaluate parents and introduce new elite genotype recombination [9, 10].

Determining genetic variation and combining ability are helpful breeding tools to assess the breeding potential of some populations or parents, assisting cotton breeders in using the right breeding techniques [11, 12]. The concept of combining ability is a helpful objective in breeding processes to compare the performance of lines in hybrid combinations. The ability of cultivars to combine with one another through hybridization to pass on advantageous genes to their offspring is referred to as combining ability. Many investigators studied heterosis by mid -parent and better parent aspects, and general and specific combining abilities among all studied genetic materials and indicated that the magnitude of heterosis versus mid-parent and better parent were significant for most studied traits [2, 11, 13–19]. It was revealed that the degree of specific combining ability (SCA) variance was greater than that of general combining ability (GCA) in respect of number of days to first flower [20]. The study of the gene action for earliness traits revealed that, with the exception of the first fruiting node, additive gene action was shown to be significantly more powerful than non-additive gene action [21]. A recent study revealed that the mean squares of SCA were greater than those of GCA for all studied traits, and as a result, the σ^2^D estimates were higher than those of σ^2^A ones for these traits. Additionally, they demonstrated that the cotton variety "Giza 90" was a good general combiner for the first fruiting node [22]. Xiaoquan et al*.* [23] studied the heterosis versus mid- parent and better parent for fiber quality traits, they found that the range of heterosis was from -3.90 to 27.29% for fiber length, -6.37 to 35.93% for fiber strength, and -0.42 to 3.53% for fiber uniformity. Jenkins et al. [24] demonstrated that the additive variances were considerably greater than the dominance variances towards plant height, the number of fruiting branches, and the earliest traits. Based on a line × tester mating design, it is possible to evaluate the genetic factors influencing yield and quality attributes in superior parents, and crossings [25].

The main objectives of this study were: 1) to use line × tester analysis to assess the combining abilities, GCA and SCA, among different cotton genotypes; 2) to identify genetic variation components for earliness, yield characteristics, and fiber quality variables, as well as heritability; 3) to ascertain if hybrid breeding may take advantage of genotype variation to improve yield and quality parameters depending on their best GCA and SCA characteristics; and 3) to investigate the nature of gene activity. Moreover, improving cotton production and fiber properties by developing cotton crosses with varied genetic origins will offer helpful and reliable knowledge for cotton breeding programs.

## Materials and methods

**Fig. 1:**
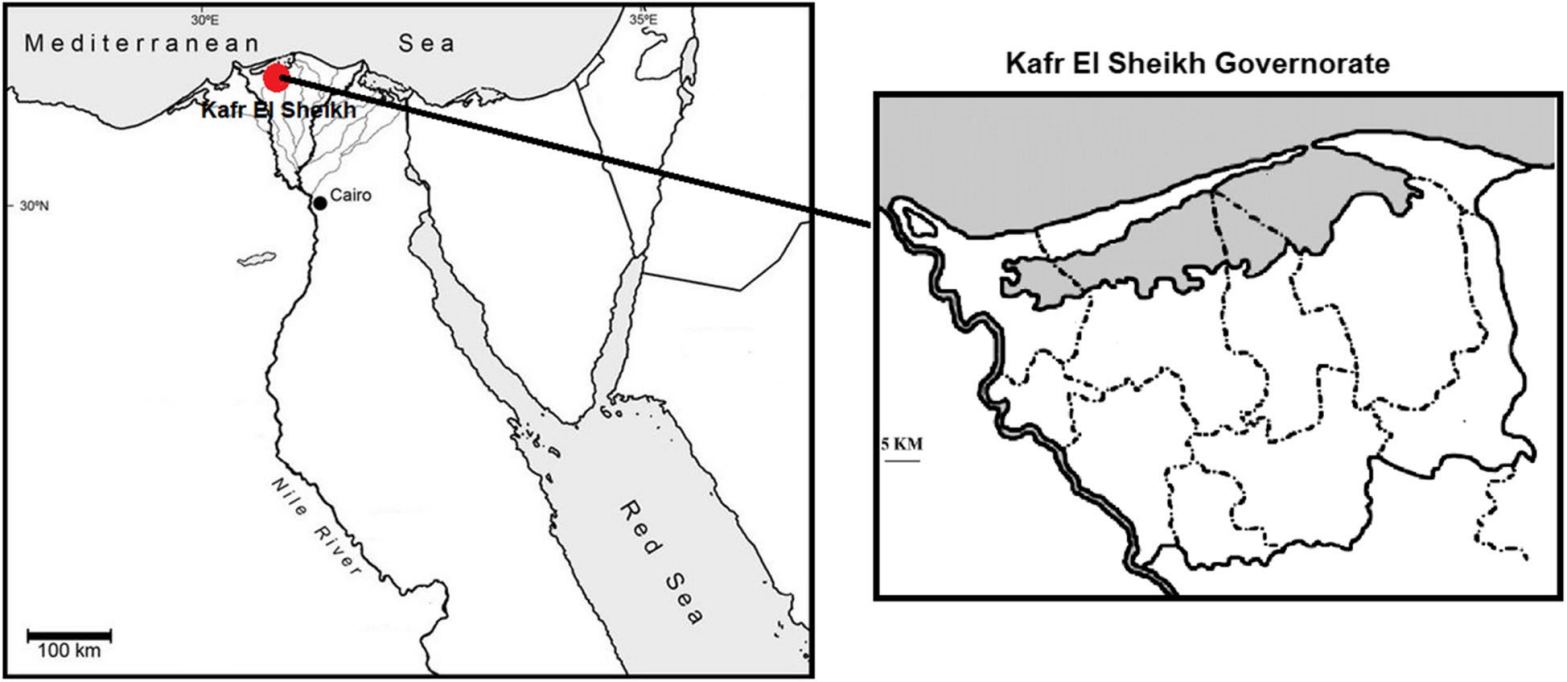
location of the experiment; at Sakha Experimental Station, Agriculture Research Center, Kafr El-Sheikh government, Egypt, (31°05′36"N, 30°56′40"E)

Twelve cotton genotypes from six distinct origins were the genetic resources utilized in the current study. These twelve cotton genotypes' origin, pedigree, and characteristics are shown in Table 1. The selfed seeds of these genotypes were kindly supported by the Cotton Breeding and Cotton Maintenance Departments, Cotton Research Institute, Agriculture Research Center, Giza, Egypt. The present investigation was carried out during the three growing seasons from 2015 to 2017 at Sakha Experimental Station, Agriculture Research Center, Kafr El-Sheikh government, Egypt, (31°05′36"N, 30°56′40"E). Sakha Agriculture Research Station, Egypt (Fig. 1) is an example of an ancient Nile Valley clay soil, which is made up of 49.97% clay, 16.97% sand, and 33.06% silt.

**Table 1:** Origin, pedigree, and characterization for the twelve parental cotton genotypes utilize in this study

No	Genotypes	Origin	Pedigree	Characterization
Lines				
L_1_	**Suvin**	India	Sujata x Vincent	Long staple variety had high lint percentage and earliness
L_2_	**Giza 89**	Egypt	(Giza 75 × 6022)	Long staple variety had early maturity and high amount of bolls/plant
L_3_	**Giza 94**	Egypt	10,229 × Giza 86	Long staple variety had good fiber quality and high yield and lint %
L_4_	**Giza 96**	Egypt	[Giza 84 x ((Giza 70 × Giza 51B) x S_6_))]	Extra-long staple variety had high yield and lint %. Also, high fiber length and strength
L_5_	**Pima S**_**6**_	USA	5934–23-2 × 615,903–98-4–4	Long staple variety, had high lint % and lint index
L_6_	**CB .58**	USA	Unknown	Genotype had high yield and lint %
L_7_	**TNB**	Australia	Unknown	Extra-long staple variety had high lint % and earliness
L_8_	**Giza 70**	Egypt	Giza 59A x Giza 51B	Extra-long staple variety had high fiber length and fiber strength
Testers				
T_1_	**Giza 86**	Egypt	Giza 75 × Giza 81	Long staple cotton variety had high yield
T_2_	**Karshenky**	Russia	Unknown	Long staple cotton variety High early maturity
T_3_	**Giza 93**	Egypt	Giza 77 × Pima S_6_	Extra-long staple variety had high fiber fineness and strength. late maturity
T_4_	**BBB**	Greece	Unknown	Long staple variety had high boll weight and lint %

The selfed seeds of twelve cotton genotypes were crossed using a line x tester mating design with four testers and eight lines to generate thirty-two cotton hybrids. According to Kempthorne [15], the eight female parents and four male testers were crossed during the 2015 growing season to create 32 F1 top crosses. In the growing seasons of 2016 and 2017, the selfed seeds of the twelve parents and their 32 F1 hybrids were planted in a randomized complete blocks design (RCBD) with three replications each replicate consisted of one row for both parents and their F1 crosses. Each row was 4.0 m long; the distance between rows was 0.7 m and within plants was 0.4 m to insure ten plants per row. Hills were thinned to keep a constant stand of one plant per hill at the seedlings stage. The recommended agricultural practices were applied at the proper time.

### Data recording

Data were recorded on ten guarded plants for earliness, growth habit, cotton yield, and fiber traits *i.e.*, first fruiting nods (FFN), number of fruiting branches per plant (No. FB/P), plant height (PH), seed cotton yield tone/hectar (SCY.t/ha), lint cotton yield tone/ hectar (LCY.t /ha), seed index (SI) in gram, number of open bolls per fruiting branches (No.B/FB), lint index (LI) and number of seeds/boll (No. S/B).

Fiber parameters were recorded in the laboratories of the Cotton Technology Research Division, Cotton Research Institute, Agriculture Research Center, Egypt, in accordance with the standard method of the American Society for Testing Materials Designation (American Society for Testing Materials, 1998) at 21 °C ± 1 °C and 65% ± 2% relative humidity. Micronaire reading (MIC) was used to express fiber fineness. Fiber strength (FS) was measured by using a Pressley instrument at zero gauge (g/tex). Fiber strength is closely related to yarn and fabric strength and spinning efficiency. Fiber length at 2.5% (FL, mm) was estimated as the space in mm spanned by 2.5% of the fibers as recorded on a digital fibro graph. The uniformity ratio (UR%) was calculated in accordance with the following equation:

$$UR\%=\frac{50\% Span length}{2.5\% Span length} X100$$

### Statistical analysis

The recorded data were analyzed using Steel and Torrie's method of variance analysis [26] to determine significant variations among cotton genotypes. For a better understanding of the link between the examined traits, Pearson's correlation was used [27, 28]. across mean performances, whereas path analysis was implemented to detect the direct and indirect effect of studied characters. All figures including cluster, correlation, and path analysis diagrams were performed using the computer software R version 4.03.

## Results

### The relationship among lines and testers (parent genotypes)

**Fig. 2:**
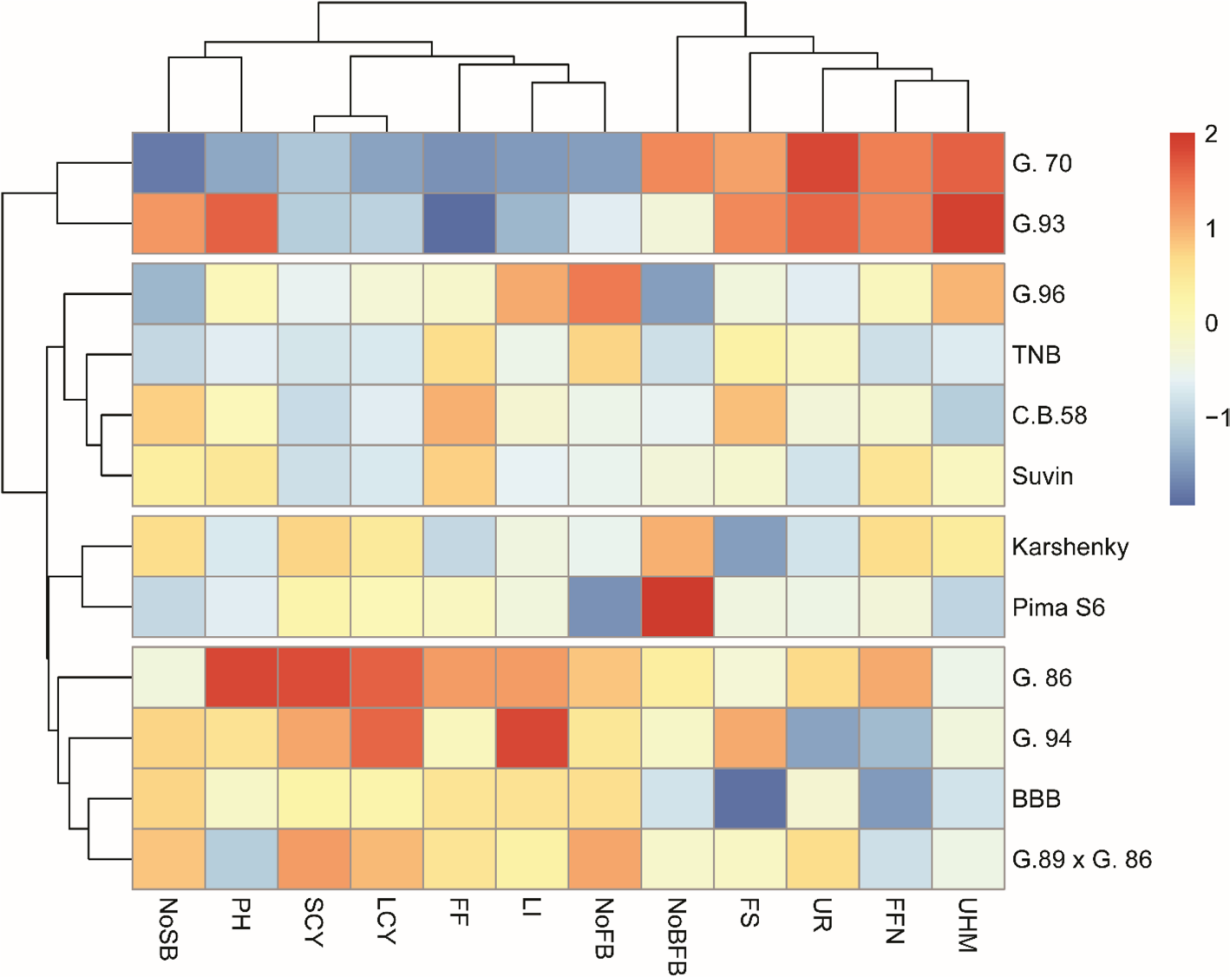
Dendrogram based on yield dissimilarity coefficients and yield component assessed throughout two years of investigation on twelve parental cotton genotypes

Twelve studied traits were used to perform the cluster analysis using Euclidean distance via the unweighted paired group method with arithmetic average (UPGMA). This cluster was basically performed depending on Jaccard similarity coefficient of 12 cotton genotypes based on yield and yield components traits across two years of study as shown in Table 2. A dendrogram was conducted using matrix data of dissimilarity coefficients as shown in Fig. 2. Two clusters were identified on the dendrogram among the twelve ancestral cotton genotypes. Ten parents made up Cluster I, which was separated into three subclusters. The first subcluster's parents were Giza 96, TNB, C.B. 58, and Suvin, while the second subcluster's parents were Karshenky and PimaS6, and the third subcluster included the most related Egyptian parents (Giza 96, Giza 94, BBB, and Giza 89 X Giza 89). The second Cluster consisted of two parents including Giza 70, and Giza 93). It is predicted that genotypes clustered inside the same cluster (intra cluster) will have a higher degree of genetic similarity than genotypes grouped within other clusters (inter cluster).

**Table 2:** Jaccard similarity coefficient of 12 cotton genotypes based on yield and yield competent traits across two years of study

	**BBB**	**C.B.58**	**G. 86**	**G. 94**	**G.89 × G. 86**	**G.93**	**G.96**	**Karshenky**	**Pima S6**	**Suvin**
C.B.58	0.0323									
G. 86	0.1106	0.1154								
G. 94	0.0545	0.0583	0.0725							
G.89 × G. 86	0.0602	0.0820	0.1381	0.0911						
G.93	0.1197	0.0939	0.0580	0.0919	0.1583					
G.96	0.0350	0.0358	0.1102	0.0631	0.0794	0.1115				
Karshenky	0.0535	0.0614	0.1414	0.0896	0.0446	0.1344	0.0709			
Pima S6	0.0565	0.0590	0.1456	0.0978	0.0582	0.1421	0.0706	0.0293		
Suvin	0.0572	0.0319	0.0929	0.0384	0.1033	0.0714	0.0526	0.0752	0.0809	
TNB	0.0443	0.0510	0.1363	0.0886	0.0444	0.1349	0.0525	0.0412	0.0318	0.0734

### Analysis of variance

The analysis of variance for each year between all the studied traits are presented in Table 3. All studied traits highly significantly affected by each of genotype, parents, and crosses except for No.S/B and UR. The interaction between parents and crosses had a significant influence of all of studied traits except for No.S/B, UR, and UHM in both seasons of study. The lines effect was highly significant of all studied characters except for No.B/FB and UR, whereas tester had significant effect on all traits except for LI (in the second season) and No.S/B, and UR in both seasons of study. All studied characters, except for UHM and UR, were significantly influenced by various interactions of parents x crosses and lines x testers during the two seasons of study.

**Table 3:** Analysis of variance (ANOVA) for earliness, growth, yield and fiber quality traits during two growing seasons

S.O.V	d.f	SCY.ton/ha		LCY.ton/ha		No.B/FB		LI		NO. S/B	
		**Y1**	**Y2**	**Y1**	**Y2**	**Y1**	**Y2**	**Y1**	**Y2**	**Y1**	**Y2**
Replications	2	1.78**	8.58**	1.23*	3.87*	2.23*	5.41*	2.34	2.64	11.22	6.036
Genotypes (G)	43	1.23**	2.65**	1.33*	3.53*	0.52*	0.59*	0.42*	0.86**	2.41	6.19
Parents (P)	11	0.36**	0.41*	0.05**	0.06**	0.09**	0.51*	0.25*	0.41*	2.45	0.55
Crosses (C)	31	1.58**	3.50**	1.81**	4.83**	0.22**	2.58*	0.49**	1.18*	2.28	26.93
P x C	1	0.04**	1.12**	0.33**	1.34*	0.67*	0.55**	0.30*	0.0034*	2.41	7.52
Lines (L)	7	0.72**	2.42**	1.44**	4.33**	0.66*	0.49	0.21**	0.28**	2.26	6.21
Testers (T)	3	7.08**	10.01**	4.01**	7.64**	2.32*	2.34**	4.75**	5.70	11.25	26.68
L x T	21	1.08**	2.93**	1.63**	4.59**	0.44**	0.31**	0.27**	0.51**	1.19	5.22
Error	86	1.20	2.16	1.34	3.29	0.74	0.34	0.53	0.52	2.86	6.11
**S.O.V**	**d.f**	**FFN/P**		**FL**		**PH**		**UR%**		**FF**	
		**Y1**	**Y2**	**Y1**	**Y2**	**Y1**	**Y2**	Y1	Y2	Y1	Y2
Replications	2	0.007	0.28	0.1	0.09	48.05	54.74	6.13	1.41	0.01	0.01
Genotypes (G)	43	1.36**	1.27**	9.92**	4.25**	950.2**	917.3**	0.74	0.62	0.21**	0.25**
Parents (P)	11	1.14**	1.27**	20.45**	8.16**	1079.6**	1088.6**	0.69	0.32	0.33**	0.23**
Crosses (C)	31	1.20**	1.25**	6.31**	2.87**	931.80**	839.90**	0.25	2.86	0.14**	0.23**
P x C	1	8.98**	1.82**	5.94**	3.94**	98.54**	1430.7**	0.78	0.653	1.07**	1.03**
Lines (L)	7	1.25**	2.70**	8.46**	1.61**	571.5**	828.6**	0.38	0.71	0.28**	0.28**
Testers (T)	3	3.22**	1.28**	6.20**	5.90**	3305.1**	1787.6**	3.39	1.75	0.12**	0.17**
L x T	21	0.89**	0.76**	5.61**	2.86**	712.9**	708.3**	0.54	0.48	0.10**	0.22**
Error	86	0.06	0.07	0.27	0.03	37.62	35.31	0.94	0.82	0.004	0.002
**S.O.V**	**d.f**	**F.S**		**UHM**							
		**Y1**	**Y2**	**Y1**	**Y2**						
Replications	2	0.01	0.02	0.99	0.02						
Genotypes (G)	43	0.73**	0.82**	2.28**	4.75**						
Parents (P)	11	1.19**	1.73**	4.79**	4.88**						
Crosses (C)	31	0.56**	0.39**	1.45**	4.86**						
P x C	1	0.82**	4.10**	0.23	0.02						
Lines (L)	7	0.85**	0.34**	2.65**	12.35**						
Testers (T)	3	0.05**	1.07**	2.22**	7.56**						
L x T	21	0.54**	0.30**	0.94**	1.97 **						
Error	86	0.01	0.01	0.37	0.85						

^*^and **: significant at 5% and 1% levels of probability, LCY. ton/ha., lint cotton yield; SCY. ton/ha, seed cotton yield; PH, plant height; No.B/P, number of bolls per plant; No. FB/P, Number of fruiting branches per plant; No.S/B, Number of seeds per boll; LI, lint index; FF, fiber fineness; FS,fiber strength; UR%, uniformity ratio; UHM, upper half means; FFN/P, First Fruiting Node

### Mean performance among all parental genotypes and F1 crosses

General performance among all parental genotypes and F1 crosses is shown in Table 4. For the mean performance among the female parental genotypes, Suvin recorded the highest overall means of SCY.K/Fad, LCY.K/Fad, PH and FF, maximum mean values of each of LCY.t /ha (1.33, and 1.37), SCY.t/ha (3.7, and 3.19), PH (221.37, and 212.58 cm), and FF (4.33, and 4.31) in both growing seasons. Likewise, highest mean values of the UR (89, and 89.10), FFN/P (7.89, and 7.84), FS (11.12, and 11.13), and UHM (35.89, and 35.79) were achieved by G.94. For No. B/FB, and No. S/B G.89 × G. 86 Exhibited the highest values in both seasons. TNB genotype recorded the greatest values of LI, while Giza 70 indicated the largest number of No. FB/P. In respect to mean performance of tester (pollinators) genotypes, G.86 recorded the highest mean values for SCY.t/ha (3.16, and 3.93), LCY.t/ha (116, and 1.47) No. B/FB (4.62, and 4.31), LI (5.48, and 5.89), No. S/B (20.53, and 21.61), and PH (221.37, and 212.58). While the tester Kar. recorded the highest F.F. (4.28, and4.02), whereas BBB possessed highest mean values for FFN (7.93, and 7.69), No.FB/P (19.19, and 22.33), FS (11.00, and 11.22), and UHM (35.45, and 35.30).

**Table 4:** The mean performances of eight parental lines and four testers for earliness, growth habit, yield and fiber quality traits for two years using line x tester hybrids of cotton.

Genotypes	SCY. ton/ha.		LCY.ton/ha.		No. B/FB		LI		No. S/B		UR%	
	**Lines**											
Suvin	3.7	3.19	1.33	1.37	3.60	3.97	6.58	6.15	19.39	20.45	88.22	87.60
G.89 x G. 86	3.08	3.2	1.18	1.21	3.99	3.99	5.45	5.39	20.40	21.31	86.94	86.74
G. 94	2.97	3.05	1.1	1.14	3.17	3.44	4.81	4.97	20.95	19.55	89.00	89.10
G.96	2.77	3.09	1.02	1.15	2.88	3.46	6.17	6.18	20.49	18.02	87.43	87.47
Pima S6	2.91	3.12	1.08	1.16	3.17	3.33	5.29	5.39	20.15	17.85	86.94	87.21
C.B.58	3.24	3.33	1.22	1.25	3.27	3.57	5.95	5.96	20.60	16.76	88.20	86.25
TNB	2.76	2.79	1.37	1.18	3.30	3.77	7.06	7.16	20.49	19.52	86.43	86.45
G. 70	3.6	3.66	1.04	1.04	2.48	3.00	6.48	5.95	18.47	18.93	87.10	88.51
*Lines average*	3.13	3.17	1.16	1.18	3.23	3.57	5.97	5.89	20.12	19.05	87.53	87.42
	**Tester**											
G. 86	3.16	3.93	1.16	1.47	4.62	4.43	5.48	5.89	20.53	21.61	87.24	87.52
Kar.	2.94	3.52	1.1	1.29	3.02	3.21	5.54	5.23	18.82	17.14	87.37	87.34
G.93	2.6	2.69	0.98	1.02	2.85	3.62	5.38	5.19	18.81	18.65	87.58	87.94
BBB	2.64	2.74	0.96	0.99	4.19	4.23	4.65	4.55	17.97	18.08	89.21	89.01
*Tester average*	2.835	3.22	1.05	1.1925	3.67	3.87	5.26	5.22	19.03	18.87	87.85	87.95
Lsd_0.05_	0.91	0.95	1.16	1.02	0.35	0.38	0.28	0.23	0.53	0.55	0.27	0.25
genotypes	**FFN**		**No. FB/P**		**PH**		**FF**		**FS**		**UHM**	
	**Lines**											
Suvin	7.69	7.83	21.61	22.67	221.37	212.58	4.33	4.31	10.12	10.11	32.87	32.75
G.89 x G. 86	7.46	6.55	18.97	19.05	172.78	168.33	3.64	3.67	9.37	9.22	34.01	33.87
G. 94	7.89	7.84	20.15	18.68	217.22	212.33	3.30	3.41	11.12	11.13	35.89	35.79
G.96	6.14	6.82	22.14	22.03	184.00	185.50	4.12	3.75	9.12	8.87	32.49	32.21
Pima S6	7.40	6.83	21.08	19.08	196.11	173.33	4.19	4.25	10.16	10.12	33.42	33.33
C.B.58	6.54	6.83	21.81	23.20	167.00	169.83	4.12	4.10	10.24	10.17	32.92	32.66
TNB	6.29	5.50	19.68	21.74	197.78	197.67	3.94	3.98	10.94	11.16	33.00	32.96
G. 70	7.05	6.63	22.17	24.19	186.67	151.67	3.89	3.55	10.05	9.96	34.69	34.56
*Lines average*	7.06	6.85	20.83	21.33	192.87	183.91	3.94	3.88	10.14	10.09	33.66	33.52
	**Tester**											
G. 86	6.86	6.67	17.79	16.31	187.22	190.83	3.92	3.93	10.02	9.92	32.29	32.54
Kar.	6.94	7.00	18.16	19.10	174.16	181.33	4.28	4.02	10.86	10.91	32.17	31.57
G.93	6.54	6.67	17.36	16.60	174.28	174.83	4.16	3.82	10.47	10.67	32.63	32.83
BBB	7.93	7.69	19.19	22.33	160.00	161.50	3.41	3.64	11.00	11.22	35.54	35.30
*Tester average*	7.07	7.01	18.13	18.58	173.92	177.13	3.94	3.85	10.59	10.68	33.16	33.06
**LSD**_**0.05**_	0.20	0.23	0.45	0.41	6.22	5.23	0.09	0.08	0.16	0.13	0.25	0.28

*LCY.* ***ton/ha*** Lint cotton yield, *SCY* **Ton/ha**, seed cotton yield, *PH* Plant height, *No.B/P* Number of bolls per plant, *No. FB/P* Number of fruiting branches per plant, *No.S/B* Number of seeds per boll, *LI* Lint index, *FF* Fiber fineness, *FS* Fiber strength, *UR%* Uniformity ratio, *UHM* Upper half means, *FFN/P* First Fruiting Node

The phenotypic mean performances of the 32 top crosses were differing from one season to the other season for all the studied traits as shown in Tables 5. The results showed that the cotton cross; G. 86 x (G.89 × G. 86), had the highest values for SCY.t/ha (4.84), LCY.t/ha (1.63), and No. B/F.B. (5.91) in the first season. While in the second season the cotton cross G.93 × Suvin recorded the highest SCY.t/ha (4.14), LCY.t/ha (1.73) No. B/F.B. (6.66). The cross Kar. x TNB recorded higher values for No. S./B. (21.29, and 22.34) in both growing seasons, respectively. For UR%, the cross Kar. x Suvin exhibited the highest value of 89.50 in the first season whereas in the second season BBB x G. 94 recorded the highest value of 88.23. As shown in cont. Table 5, The results showed that cotton crosses; BBB x TNB had the highest values for FFN (7.77 and 7.04), number of fruiting branch/plant (No. FB/P) (22.44, and 23.38), and plant height (PH) (239.82 and 210.41 cm), in the two growing seasons, respectively. The cross BBB x Giza 94 indicated remarkable values for fiber strength (11.04, and 11.20) while for FF greatest values were obtained from cotton cross Kar. x (G.89 × G. 86) (4.64, and 4.44) in both seasons. This may be related to the two parents belonging to the long staple category as shown in Table 4. All the thirty-two cotton crosses showed fiber lengths higher than 31 mm and less than 34 mm this means that these crosses belonged to the long staple category except two crosses Karshenky x Giza 94 and Karshenky x Giza 96 had fiber lengths of 35.00 and 35.80 mm coupled with 10.72 and 10.30 for fiber strength and 4.15 and 4.00 for micronaire reading. While all 32 F1 cotton crosses recorded good values for micronaire reading, fiber strength, and fiber length.

**Table 5:** Mean performance of hybrids for earliness, growth habit, yield and fiber quality traits for two years using line x tester hybrids of cotton

Genotypes	SCY. ton/ha		LCYton/ha		No. B/FB		LI		No. S/B		UR%		FFN		No. F.B/P		PH		FF		FS		UHM	
	**Y1**	**Y2**	**Y1**	**Y2**	**Y1**	**Y2**	**Y1**	**Y2**	**Y1**	**Y2**	**Y1**	**Y2**	**Y1**	**Y2**	**Y1**	**Y2**	**Y1**	**Y2**	**Y1**	**Y2**	**Y1**	**Y2**	**Y1**	**Y2**
**G. 86 × Suvin**	3.42	3.54	1.27	1.34	5.12	5.06	7.94	7.05	18.26	19.53	86.83	87.77	6.67	6.83	19.74	19.47	185.00	176.04	4.38	3.51	10.35	10.69	33.30	33.33
**G. 86 x (G.89 × G. 86)**	4.84	4.17	1.63	1.55	6.66	5.26	7.06	6.91	17.63	18.78	86.83	87.50	5.90	5.67	21.13	19.27	178.37	176.54	4.20	4.27	9.91	11.05	32.03	33.70
**G. 86 × G. 94**	3.19	3.19	1.15	1.16	3.43	4.50	6.96	5.87	17.67	19.80	86.80	86.93	5.78	6.17	19.83	20.07	167.22	171.67	3.90	3.69	10.55	11.04	33.43	34.20
**G. 86 × G. 96**	3.23	3.75	1.19	1.37	4.00	3.48	6.75	6.49	15.12	18.84	87.63	86.83	5.67	5.60	21.42	18.80	180.00	178.37	4.25	4.03	9.87	10.28	33.27	32.23
**G. 86 × Pima S6**	3.59	3.84	1.31	1.41	4.34	4.91	6.84	6.57	17.48	17.50	86.87	86.30	5.74	6.47	21.02	21.30	202.78	183.85	4.31	4.72	10.07	10.38	33.23	32.67
**G. 86 × C.B.58**	3.11	2.9	1.13	1.05	4.34	4.93	7.23	6.56	16.06	18.59	87.07	86.07	6.50	6.30	20.97	20.57	196.72	213.83	4.38	4.40	10.41	10.03	32.97	31.83
**G. 86 × TNB**	3.24	3.37	1.24	1.26	4.88	4.34	6.40	6.40	20.02	19.82	89.13	87.43	6.78	7.14	21.93	22.19	201.33	190.78	4.16	4.34	10.50	10.36	33.33	33.63
**G. 86 × G.70**	2.76	3.11	1.05	1.18	3.18	3.94	6.07	5.74	21.28	21.08	86.93	85.90	6.66	7.29	21.73	22.83	180.00	173.48	3.93	3.74	9.67	10.74	32.83	33.13
**Kar. x Suvin**	3.18	3.34	1.26	1.3	5.12	4.64	6.63	6.70	18.43	20.79	89.50	87.13	6.11	5.83	20.95	20.56	165.00	156.52	3.90	4.18	10.92	10.58	34.30	33.63
**Kar. x (G.89 × G. 86)**	3.05	2.9	1.14	1.1	3.69	3.61	6.32	6.01	19.88	22.01	86.73	86.90	5.78	6.50	21.45	20.35	173.89	142.83	4.64	4.44	9.70	10.58	33.33	34.37
**Kar. x G. 94**	3.38	3.29	1.26	1.22	4.95	5.14	5.82	5.74	17.64	17.78	87.80	87.43	5.56	6.99	21.53	22.98	176.39	170.00	4.15	4.00	10.47	10.97	35.00	35.80
**Kar. x G. 96**	3.1	3.2	1.14	1.13	3.72	4.86	6.80	6.16	15.15	16.74	87.40	87.60	6.33	5.50	19.73	18.86	186.16	164.17	3.91	3.98	10.10	10.51	35.47	36.10
**Kar. x Pima S6**	3.23	3.33	1.16	1.18	3.71	4.17	6.30	6.32	19.91	21.32	86.77	86.70	5.78	6.23	22.00	21.33	161.87	171.67	4.09	4.31	9.80	9.87	32.73	32.57
**Kar. x C.B.58**	2.53	3.06	0.95	1.11	3.43	4.89	5.50	5.76	18.09	18.79	86.03	86.47	5.89	6.27	18.48	19.32	173.14	170.91	4.28	4.38	9.73	10.09	32.20	33.03
**Kar. x TNB**	2.45	2.56	0.88	0.92	3.85	5.56	6.16	5.79	21.29	22.34	86.83	86.57	6.85	7.67	19.42	23.52	163.04	151.50	4.12	3.75	10.20	11.06	33.73	32.60
**Kar. x G.70**	2.3	2.64	0.84	0.96	2.93	3.73	5.72	5.89	20.40	19.20	86.37	87.67	6.89	7.24	19.48	19.46	176.67	173.33	3.87	4.19	9.74	10.42	33.33	34.77
**G.93 × Suvin**	2.64	2.92	0.97	1.06	4.64	5.91	7.99	6.51	16.95	20.59	86.33	87.33	6.56	7.09	19.36	22.11	180.71	174.50	4.21	4.44	10.04	10.45	33.90	34.67
**G.93 x (G.89 × G. 86)**	3.53	4.14	1.5	1.73	3.77	5.58	6.56	6.12	18.91	20.76	86.97	87.80	6.77	6.67	19.36	20.62	198.16	190.07	4.36	4.33	10.35	10.84	33.33	33.17
**G.93 × G. 94**	2.38	3.16	0.87	1.17	3.38	2.88	5.73	5.42	19.82	20.60	88.40	86.80	5.33	5.83	21.45	18.43	173.89	166.71	3.91	3.77	9.50	10.94	33.23	32.97
**G.93 × G. 96**	2.22	2.62	0.81	0.95	3.88	3.44	6.51	6.31	20.54	21.40	87.03	87.53	6.98	6.50	20.06	19.37	175.56	164.17	3.99	3.94	10.94	11.10	32.90	34.10
**G.93 × Pima S6**	2.12	2.5	0.77	0.93	4.42	3.75	6.48	5.92	19.36	21.91	86.50	85.73	6.78	7.17	20.02	21.82	165.85	160.00	4.47	4.12	10.00	10.27	32.87	30.97
**G.93 × C.B.58**	2.13	2.64	0.78	0.96	4.24	5.00	6.42	5.99	19.14	22.28	86.07	85.53	7.11	7.83	20.85	21.76	196.60	177.45	4.24	4.17	9.87	10.80	32.73	30.87
**G.93 × TNB**	2.66	2.98	0.91	1.09	4.49	5.34	6.49	6.03	18.82	20.21	87.00	86.93	7.56	8.00	20.02	21.10	187.63	194.04	4.15	3.91	10.91	10.47	33.53	32.47
**G.93 × G.70**	2.97	3.2	1.08	1.14	3.08	3.65	5.53	5.66	18.90	19.31	86.50	87.20	5.78	6.61	19.60	19.34	162.93	164.17	4.29	3.95	9.48	10.53	33.43	33.40
**BBB x Suvin**	3.62	3.85	1.39	1.44	4.30	4.51	7.67	6.97	17.45	13.22	86.60	86.60	6.67	6.50	20.04	22.11	180.10	172.44	4.33	4.08	9.74	11.13	32.80	33.57
**BBB x (G.89 × G. 86)**	2.92	3.06	1.163	1.18	3.14	4.71	6.64	5.90	20.68	17.18	87.07	86.77	5.83	6.38	20.31	21.08	200.71	161.61	4.62	3.97	9.93	11.03	33.73	34.27
**BBB x G. 94**	3.24	3.48	1.25	1.3	3.20	4.14	6.22	5.99	17.77	15.42	88.47	88.23	7.44	5.73	18.62	21.51	200.56	145.58	3.62	3.86	11.04	11.20	34.40	34.93
**BBB x G. 96**	3.44	3.7	1.31	1.42	2.95	4.75	5.92	6.09	16.70	17.29	88.03	87.33	6.88	6.50	19.60	22.89	218.00	179.20	3.74	3.86	10.23	11.20	34.43	34.27
**BBB x Pima S6**	4.22	4.12	1.61	1.51	2.99	3.03	6.03	5.71	17.42	19.11	87.20	86.23	7.30	7.04	21.02	22.01	198.89	166.67	4.17	3.76	9.77	11.20	33.50	31.70
**BBB x C.B.58**	3.68	3.96	1.42	1.48	3.45	3.80	6.72	6.33	17.99	18.11	86.43	86.20	7.56	7.17	19.33	20.57	165.56	173.77	4.28	3.90	9.58	10.97	32.30	31.97
**BBB x TNB**	2.94	3.53	1.13	1.34	3.84	4.08	6.78	6.67	17.04	18.30	87.27	87.37	7.77	7.07	22.44	23.38	239.82	210.41	4.12	3.85	10.20	10.90	34.23	32.80
**BBB x G.70**	2.98	2.86	1.37	1.31	2.77	4.21	5.46	5.42	19.84	20.76	87.53	87.93	7.00	6.73	19.17	19.55	195.00	209.83	4.22	4.32	10.03	10.83	33.97	33.77
**LSD **_**0.05**_	**0.50**	**0.45**	**0.43**	**0.45**	**0.18**	**0.20**	**0.11**	**0.10**	**0.43**	**0.40**	**0.26**	**0.23**	0.41	0.41	0.29	0.45	4.95	4.64	0.86	0.77	0.13	0.16	0.99	0.17

LCY. ton/ha., lint cotton yield; SCY. ton/ha, seed cotton yield; No. FB/P, Number of fruiting branches per plant; LI, lint index; No.S/B, Number of seeds per boll; UR%, uniformity ratio. UHM, upper half means; FS,fiber strength; FF, fiber fineness.; PH, plant height; FFN/P, First Fruiting Node; No.B/P, number of bolls per plant

### General combining ability

Estimates of general combining ability effects of lines and testers for all the studied traits in each year are presented in Table 6. For GCA estimates of SCY.t/ha, No. B/F. B, maximum positive GCA effect was recorded by Pima S6 (0.56, and 86), and (0.56, and 0.30) respectively, while Suvin recorded the largest value of LCY. t/ha (0.99, and 0.72). For testers, the highest positive effect of GCA for SCY. t/ha., LCY. t/ha, No. B/FB and LI were obtained by BBB (1.14, and 0.97), (1.45, and 1.54), (0.38, and 0.38), and (0.52, and 0.42) respectively. whereas maximum negative GCA effect was recorded by G.93 (- 0.95, and -1.35), (-1.79, and -1.19), (-0.38) and (-0.53) for SCY. t/ha, LCY. t/ha, No. B/FB and L.I., respectively. With respect to No. S/B, and LI highest positive and significant GCA effect among parental lines was recorded by Suvin, while G.96 (-0.115, and 0.791) and G.94 (-0.481, and -1.274) recorded the highest negative and significant GCA effects. Two parental lines showed a good general combiner for UR%, namely G. 70 (0.252) and TNB (0.399), while G.89 × G. 86 (-0.28, and -0.377) showed a negative GCA effect for UR%, indicating unfavorable genotypes for general combining ability.

**Table 6:** General combining ability effects of parental genotypes for earliness, growth habit, yield and fiber quality traits during two years using line x tester hybrids of cotton

Genotypes	SCY. ton/ha			LCY. ton/ha				No. B/FB				LI				No. S/B				UR				
	**Lines**																							
Suvin	0.461**	0.66**		0.99*		0.72*		0.056**		-0.01*		0.27**		0.49*		0.55**		0.85**		-0.03		-0.17		
G.89 × G. 86	-0.538**	-0.34*		-0.2		-0.48*		-0.301**		-0.38*		0.06		-0.64*		0.57*		0.67*		-0.28		-0.37		
G. 94	0.122**	0.22**		0.23		0.11*		0.016*		-0.10*		0.03		0.07*		-0.48*		-1.27*		-0.08		-0.002		
G.96	0.221**	0.08*		0.03		0.16**		-0.15**		0.18*		-0.11		-0.79**		0.34*		-0.47*		0.04		-0.11		
Pima S6	0.561**	0.86*		0.90*		0.61**		0.58**		0.30*		0.015*		-0.64*		-0.31*		-0.40*		-0.07		0.03		
C.B.58	-0.25*	-0.28*		-0.29*		-0.38**		0.263**		0.03		-0.12		-0.81*		-0.33*		-0.10*		0.23		0.24		
TNB	-0.335**	-0.38*		-0.57*		-0.37*		-0.025**		-0.03*		-0.02		-0.75**		-0.35*		0.07*		-0.12		0.399		
G. 70	-0.23*	-0.82*		-1.02*		-0.36*		-0.238**		-0.014		-0.11		-0.93*		0.001		0.64*		0.25		-0.01		
	**Tester**																							
G. 86	0.32**	0.55*		0.56*		0.35*		0.03*		-0.17*		-0.14*		-0.68*		1.02**		0.73**		-0.51**		0.10		
Kar	-0.52*	-0.17**		-0.22*		-0.70**		-0.04*		-0.07		0.15*		0.22**		-0.41*		-1.56**		0.28*		0.08		
G.93	-0.95**	-1.35**		-1.79**		-1.19**		-0.38*		-0.14		-0.53**		-0.11		-0.24*		0.27*		-0.05		0.22*		
BBB	1.14*	0.97**		1.45**		1.54**		0.38*		0.38*		0.52**		0.42**		-0.36**		0.56**		0.29**		-0.40**		
Genotypes	FFN				No. FB/P				PH				FF				FS				UHM			
	**Lines**																							
Suvin	0.03		-0.08		0.17		-0.29**		-6.90**		-4.38*		0.06**		-0.01		0.15**		0.03		0.18		0.41	
G.89 × G. 86	-0.40**		-0.34**		-0.56**		0.25**		3.17		-6.49**		0.16**		0.18**		-0.14**		0.19**		-0.29		0.49	
G. 94	-0.45**		-0.46**		-0.14		0.05		-5.10**		-10.77**		-0.25**		-0.24**		0.28**		0.20**		0.62**		1.09**	
G.96	-0.01		-0.62**		-0.91**		-0.11*		5.32**		-2.78		-0.17**		-0.11**		0.17**		0.09**		0.37*		1.04**	
Pima S6	-0.08		0.08		0.72**		0.70**		-2.26		-3.71*		0.12**		0.16**		-0.20**		-0.25**		-0.32		-1.41**	
C.B.58	0.29**		0.25**		-0.34*		-0.41**		-1.61		9.74**		0.15**		0.15**		-0.22**		-0.21**		-0.85**		-1.46**	
TNB	0.52**		0.83**		1.66**		0.14**		13.35**		12.43**		-0.01		-0.10**		0.34**		0.01		0.31		-0.51	
G. 70	0.11		0.33**		-0.60**		-0.32**		-5.96**		5.95**		-0.07**		-0.02		-0.38**		-0.05		-0.01		0.38	
	**Tester**																							
G. 86	-0.26**		-0.21**		-0.33**		0.66**		1.82		8.72**		0.05**		0.02*		0.05**		-0.11**		-035**		-0.30	
Kar	-0.33**		-0.11*		-0.09		0.07		-12.59**		-11.64**		-0.10**		0.09**		-0.03*		-0.18**		0.24		0.72**	
G.93	0.13*		0.32**		-0.32**		-0.23**		-4.44**		-0.37		0.06**		0.01		0.02		-0.01		-0.16		-0.56*	
BBB	0.46**		-0.01		0.47**		-0.50**		15.22**		3.19**		-0.01		-0.12**		-0.05**		0.30**		0.27*		0.14	

^*^and **: significant at 5% and 1% levels of probability,LCY. ton/ha., lint cotton yield; SCY. ton/ha, seed cotton yield; PH, plant height; No.B/P, number of bolls per plant; No. FB/P, Number of fruiting branches per plant; No.S/B, Number of seeds per boll; LI, lint index; FF, fiber fineness; FS,fiber strength; UR%, uniformity ratio; UHM, upper half means; FFN/P, First Fruiting Node

For fiber quality measures, the parental line G.89 × G.86 had a GCA effect of 0.16 and 0.18 for FS., respectively, while G.94 (0.62, and 1.09) for UHM. While TNB and G. 94 showed a preferable GCA effect for FS, in both seasons respectively. Data showed that the best desirable general combining ability effects for FFN/P, number of fruiting branch/plant, and plant height was TNB which had highly significant and positive GCA. While CB 58 and Pima s6 had highly significant and positive GCA for FFN and number of fruiting branch/plant. On the other hand, undesirable general combining ability effects showed highly significant and negative GCA Giza 89 × Giza 86 and Giza 94 for FFN and Suvin and Giza 94 for plant height.

The testers Giza 93 and BBB exhibited highly significant positive (desirable) general combining ability effects FFN and plant height, respectively. While the highly non-significant negative (undesirable) general combining ability affects Giza 86 and Karshenky for FFN/P; Giza 93 for fruiting branch/plant and plant height for Karshenky. For fiber quality traits the lines showed those genotypes; Giza 89 × Giza 86, Pima s6, and CB 58 had highly significant and positive GCA for micronaire read (Table 6). The two commercial varieties Giza 94 and Giza 96 had highly significant and positive GCA for fiber strength. Giza 94 has a highly significant and positive GCA and highly significant and negative GCA for fiber length each year and over two years.

### Specific combining ability

Estimates of specific combining ability effects of the 32 Egyptian cotton hybrids for all the studied traits for each year and the combined over two years are presented in Tables 7. Two hybrids Giza 86 × Suvin and Giza 86 × Giza**70** had highly significant positive SCA (desirable) effects for FFN and another two hybrids (Giza 86 × Giza 70 and Karshenky x Giza 94) exhibit highly significant positive SCA (desirable) effects for number of fruiting branch/plant. Also, five hybrids Giza 86 × CB 58, Karshenky x Giza 94, Giza 93 x (Giza 89 × Giza 86), BBB x TNB, and BBB x Giza 70 exhibited highly significant positive SCA (desirable) effects for plant height in each year and over the two years.

**Table 7:** Estimates of specific combining ability for earliness, growth habit, yield and fiber traits during two years using line x tester hybrids of cotton

Genotypes	SCY.t/ha		LCY.t/ha		No. B/FB		LI		No. S/B		UR		**FFN**		**No. FB/P**		**PH**		**FF**		**FS**		**UHM**	
	Y1	Y2	Y1	Y2	Y1	Y2	Y1	Y2	Y1	Y2	Y1	Y2	**Y1**	**Y2**	**Y1**	**Y2**	**Y1**	**Y2**	**Y1**	**Y2**	**Y1**	**Y2**	**Y1**	**Y2**
G. 86 × Suvin	0.02	-0.01	-0.16	0.13	0.31	0.20*	-0.37	0.48	-0.59	-0.69*	0.11	0.15*	0.43**	0.47**	-1.26**	-0.94**	5.48	-2.65	0.13**	-0.56**	0.03	0.09	0.07*	-0.17
G. 86 x (G.89 × G. 86)	0.03	-0.53**	-0.89**	-0.26	-0.03	0.03	-0.35*	0.23	-0.44	0.67*	-0.52*	-0.31*	0.09	-0.42**	-0.73*	-0.09	-11.20**	-0.04	-0.15**	0.00	-0.11**	0.28**	-0.72*	0.12
G. 86 × G. 94	-0.56	-0.17*	-0.40*	-0.80**	0.07*	0.11*	-0.20*	-0.36*	0.29*	0.64*	-0.23*	-0.39*	0.01	0.19	-0.35	-1.18**	-14.10**	-0.64	-0.04	-0.15**	0.11*	0.26**	-0.23	0.02
G. 86 × G. 96	0.82**	0.07*	0.16	0.90**	-0.11*	0.58	0.26*	0.54*	0.18*	-0.45*	0.48*	0.29*	-0.53**	-0.22	-0.85**	0.56**	-11.74**	-1.92	0.23**	0.056*	-0.47**	-0.37**	-0.15	-1.89**
G. 86 × Pima S6	0.71**	0.26**	0.21	0.78*	-0.05*	0.28*	0.15*	0.79*	0.28*	-0.16*	0.04	0.74*	-0.39**	-0.05	0.02	-0.65**	18.61**	4.49	0.00	0.47**	0.110*	0.07	0.50	0.99
G. 86 × C.B.58	-0.97*	0.10	-0.06	-1.16*	-0.17*	-0.70*	-0.08	0.99*	0.08*	-0.22*	-0.10*	0.46*	0.00	-0.38*	0.34	0.41**	11.89**	21.02**	0.04	0.16**	0.46**	-0.32**	0.76*	0.21
G. 86 × TNB	0.35*	0.55**	1.14**	0.58*	0.14	-0.08	0.38	0.22*	-0.05*	-0.22*	0.31*	-0.16*	0.05	-0.12	-0.03	0.824**	1.56	-4.72	-0.02	0.361**	-0.01	-0.22**	0.03	1.05*
G. 86 × G.70	-0.41*	-0.27*	0.02	-0.17	-0.16*	-0.43*	0.20	0.10*	0.23*	0.43*	-0.09	-0.78*	0.34*	0.52**	2.86*	1.07**	-0.47	-15.54**	-0.19**	-0.32**	-0.11**	0.22**	0.21	-0.34
Kar. x Suvin	0.33*	0.08	0.55*	0.79**	-0.15*	-0.24	0.50	-0.85	0.13*	2.17*	-0.64*	-0.40*	-0.06	-0.61**	-0.41	0.86**	-0.11	-1.72	-0.20**	0.04	0.68**	0.04	0.49	-0.89
Kar. x (G.89 × G. 86)	0.16*	0.75*	0.80*	0.37	0.00	-0.30*	0.17	-0.10	-0.23*	1.83*	0.08	0.03*	0.04	0.31*	0.12	0.822**	-1.10	-13.29**	-0.16**	0.099**	-0.24**	-0.12*	-0.01	-0.23
Kar. x G. 94	0.56*	1.05**	1.26***	0.74**	0.33*	0.03*	0.21*	0.25	-0.59	-1.65*	0.22*	-0.08*	-0.14	0.92**	2.32**	1.108**	9.46**	18.15**	0.35**	0.08**	0.11*	0.25**	0.74*	0.61
Kar. x G. 96	0.20*	0.44*	0.44	-0.03	-0.07	-0.06*	-0.08	-0.33	1.07	1.00	-0.51*	0.07*	0.19	-0.41**	-1.02**	-0.53**	8.82*	4.33	0.04	-0.06*	-0.15**	-0.09	0.46	0.96
Kar. x Pima S6	0.21*	0.00	-0.27	-0.10	0.12	0.41*	-0.43*	-0.38	0.86	-0.17	-0.05*	0.02	-0.29*	-0.38*	-0.19	0.92**	-7.88*	12.75**	-0.070*	-0.09**	-0.08	-0.38**	-0.59	-0.13
Kar. x C.B.58	0.31**	-0.71*	-0.78*	0.36*	-0.06	0.34*	0.16*	-0.01	-0.41*	-0.48	0.19*	-0.56*	-0.54**	-0.51**	-1.14**	-1.49**	2.73	-1.44	0.08*	0.08**	-0.05	-0.20**	-0.59	0.39
Kar. x TNB	-0.93**	-0.83**	-1.05*	-1.22*	0.08	-0.07	-0.38	-0.28	-0.37*	-0.62*	0.17*	0.30*	0.19	0.31*	1.06**	-1.10**	-22.32**	-23.54**	0.08*	-0.30**	-0.22**	0.54**	-0.21	-0.99
Kar. x G.70	-0.84**	-0.78**	-0.96**	-0.91*	-0.26**	-0.10	-0.14	-0.30	-0.46*	-0.07*	0.55*	0.63*	0.63**	0.38*	-0.74*	-0.58**	10.60 **	4.77	-0.11**	0.05	0.04	-0.04	-0.21	0.28
G.93 × Suvin	-0.36*	-0.16	-0.34	-0.68*	-0.15	-0.17*	0.05*	0.73*	0.22*	-0.69**	0.38*	0.20*	-0.08	0.20	1.37**	-0.43**	7.45*	4.99	-0.06	0.37**	-0.24**	-0.25**	0.48	1.43**
G.93 x (G.89 × G. 86)	0.88**	0.54*	0.83**	1.13**	0.18	0.55*	0.23*	0.00	0.31*	-1.17*	0.51*	0.24*	0.60**	0.04	0.61*	-0.97**	14.82**	22.67**	0.00	0.06*	0.35**	-0.02	0.38	-0.14
G.93 × G. 94	0.62**	-0.43*	-0.40*	0.85**	-0.21**	0.05	0.10*	-0.88	-0.45*	1.96*	-0.33*	0.16*	-0.82**	-0.66**	-1.99**	1.31**	-1.18	3.59	-0.04	-0.07**	-0.91**	0.06	-0.63	-0.94
G.93 × G. 96	-0.89**	-0.72**	-0.80*	-1.01**	-0.09	-0.36*	-0.11*	0.72	-0.66*	0.08*	0.12*	-0.30*	0.38*	0.15	-0.29	0.08	-9.92**	-6.94*	-0.04	-0.03	0.63**	0.33**	-0.71*	0.24
G.93 × Pima S6	-1.57**	-1.75**	-1.91**	-1.63**	-0.74**	-0.69*	-0.22*	0.63	-0.02*	-0.03	-0.33**	-0.45*	0.25	0.12	0.53	-0.76**	-12.05**	-10.18**	0.15**	-0.11**	0.07	-0.15*	-0.06	-0.44
G.93 × C.B.58	-0.38*	-0.59*	-0.63*	-0.39*	-0.14	0.14	-0.16*	0.46	-0.37*	-0.44	-0.14*	0.05*	0.21	0.62**	-1.52**	1.16**	18.03**	-6.18	-0.11**	-0.05	-0.05	0.33**	0.34	-0.49
G.93 × TNB	0.61**	0.93**	0.64*	0.64*	0.26*	0.10	-0.13*	0.54	0.85*	0.63	0.35*	-0.07*	0.43**	0.21	-1.12**	-0.21*	-5.88	7.72*	-0.05	-0.07	0.43**	-0.21**	-0.02	0.16
G.93 × G.70	1.09*	2.18*	2.62**	1.08*	0.89**	0.38*	0.24*	0.80	0.12*	-0.34*	-0.56*	0.17*	-0.93**	-0.67**	-0.63*	-0.17	-11.27**	-15.67**	0.15**	-0.10**	-0.27**	-0.09	0.20	0.20
BBB x Suvin	0.01	0.10	-0.04	-0.24*	-0.01	0.21	-0.18*	0.64	0.24*	-0.78*	0.15*	0.05*	-0.28*	-0.06	0.30	0.51**	-12.82**	-0.62	0.13**	0.14**	-0.47**	0.11*	-1.04**	-0.38
BBB x (G.89 × G. 86)	-1.06**	-0.75*	-0.75*	-1.24**	-0.15	-0.28*	-0.04*	0.87	0.36*	-1.33*	-0.07*	0.04*	-0.69**	0.08	0.00	0.243*	-2.29	-9.337**	0.320**	-0.160**	0.00	-0.143*	0.35	0.25
BBB x G. 94	-0.62*	-0.45*	-0.46*	-0.79**	-0.19*	-0.19*	-0.11	-0.02	0.74*	1.04*	0.34*	0.30*	0.95**	-0.44*	0.02	-1.24**	5.83	-21.09**	-0.27**	0.14**	0.69**	-0.58**	0.11	0.32
BBB x G. 96	-0.13	0.20	0.20	0.14	0.27	-0.16	-0.07	0.07*	-0.59*	-0.63*	-0.0	-0.05*	-0.04	0.47**	2.16**	-0.11	12.85**	4.54	-0.22**	0.03	0.00	0.13*	0.40	0.70
BBB x Pima S6	0.65*	1.49**	1.97**	0.95*	0.66*	0.00	0.50*	0.96*	-1.13	0.36*	0.34*	-0.31*	0.43**	0.312*	-0.35	0.50**	1.33	-7.06*	-0.08*	-0.35**	-0.09*	0.47**	0.15	0.42
BBB x C.B.58	1.04**	1.21*	1.47**	1.19**	0.37*	0.22*	0.07*	0.56*	0.70*	1.15*	0.05*	0.05*	0.33*	0.28	-0.73*	-0.08	-32.66**	-13.40**	-0.01	-0.196**	-0.27**	0.19**	-0.52	-0.10
BBB x TNB	-0.04	-0.66	-0.72*	0.00	-0.47*	0.05	0.14*	0.52*	-0.43*	0.21*	-0.82*	-0.07*	-0.67**	-0.39**	0.09	0.48**	26.64**	20.53**	-0.01	0.01	-0.20**	-0.10	0.25	-0.22
BBB x G.70	0.15	-1.14**	-1.68**	-0.01	-0.48*	0.15*	-0.30*	0.40*	0.11*	-0.02	0.10	-0.02	-0.04	-0.24	-1.49**	-0.32*	1.14	26.44**	0.14**	0.38**	0.35**	-0.10	0.30	-0.14
**LSD **_**0.05**_	**0.50**	**0.45**	**0.43**	**0.45**	**0.18**	**0.20**	**0.11**	**0.10**	**0.43**	**0.40**	**0.26**	**0.23**	0.29	0.29	0.60	0.21	7.04	6.82	0.06	0.07	0.13	0.10	0.70	0.68

^*^and **: significant at 5% and 1% levels of probability,LCY.K/Fad., lint cotton yield; SCY.K/Fad, seed cotton yield; No. FB/P, Number of fruiting branches per plant; LI, lint index; No.S/B, Number of seeds per boll; UR%, uniformity ratio. *and **: significant at 5% and 1% levels of probability,UHM, upper half means; FS,fiber strength; FF, fiber fineness.; PH, plant height; FFN/P, First Fruiting Node; No.B/P, number of bolls per plant

The three studied fiber quality; two cotton hybrids (Giza 86 × Giza70 and BBB x Pima S6) showed highly significant negative SCA (desirable) effects for micronaire reading, while another four hybrids Karshenky x Giza 94, G.93 × Pima S6, BBB x (Giza 89 × Giza 86) and BBB x Giza70 had highly significant positive SCA (undesirable) effects. Two hybrids Giza 86 × Giza 96 and Giza 93 × Suvin had highly significant and negative SCA (undesirable) effects and only one cross Giza 93 × Giza 96 exhibited highly significant and positive SCA (desirable) for fiber strength during each year and over the two growing seasons. Four cotton hybrids Giza 86 × Pima S6, Karshenky x Giza 96, Karshenky x Pima S6, and Giza 93 × Suvin exhibit highly significant and positive SCA (desirable) for fiber length over the two years only. While during each year the four hybrids showed non-significant SCA. Thus, the significance of GCA (variances due to lines and testers) and SCA (variances due to lines x testers) implied that both additive and non-additive types of variation were available for all the characters, yet additive genes were more important than the dominant genes because variance due to GCA was higher than that of SCA.

### Estimating the Mid-parent heterosis

Variability in genetic distance and origin was a crucial factor in creating new recombination that led to the phenomenon of heterosis. Heterosis is defined as the deviation in the performance of an F1 hybrid compared to its mid-parent or better-parent and results in improved adaptation due to the superiority of the F1 hybrid over its parents in one or more characteristics. The mid-parent heterosis was followed in the current study and showed that all thirty-two hybrids studied exhibited both positive and negative heterosis for all the twelve measured traits (Table 8). For SCY, the highest significant positive heterosis was for BBB x Pima S6 (82.63 and 79.43%) in the first and second seasons, respectively. For LCY, the highest significant heterosis values were obtained by Kar. x C.B.58 (86.04%) and Kar. x Pima S6 (85.71%), in the two seasons of the study, respectively. For No. B/FB, the two hybrids, BBB x Pima S6 and Kar. x C.B.58, scored the highest significant heterosis values of 51.97% and 82.29%, in the first and second seasons, respectively. According to LI, the two hybrids Kar. x Pima S6 (24.03%) and BBB x Pima S6 (33.65%), yielded the top two significant positive heterosis values. As regard No. S/B, the highest significant and positive heterosis were for BBB x G. 94 (32.52 and 21.72%) in the first and second seasons, respectively. For U.R., the highest significant heterosis values were obtained by G. 86 × G. 94 (2.95%) and G. 93 × G. 94 (2.53%), in the two seasons of the study, respectively. For F.F. N, the two hybrids, G. 86 × G. 94 and BBB x G. 94, scored the highest significant heterosis values of 16.51% and 19.73%, in the first and second seasons, respectively. In respect to No.F/B, the two hybrids, G.93 × G. 96 (9.63%), and G. 86 × G. 96 (12.98%), yielded the top two significant positive heterosis values. Concerning P.H., the results of heterosis versus mid-parent revealed that the G. 86 × Suvin had the highest significant values of 21.39 and 30.15% in the first and second seasons, respectively. For F.F, the two hybrids BBB x C.B.58 (8.73%) and G.93 × Suvin (20.43%) had the best performance with respect to recording the highest significant and positive heterosis, in the first and second seasons, respectively. As regard F.S, G.93 × Pima S6 (10.87%) and Kar. x G. 96 (5.38%) exhibited the highest significant positive heterosis values across the two seasons of study. For UHM, the hybrid G. 86 × G. 94 scored a positive and significant heterosis of 6.96 and 12.25% in the first and second seasons of study, respectively.

**Table 8:** Heterosis relative to mid-parent (MP) for yield and fiber traits for two years using line x tester hybrids of cotton

Cross	SCY		LCY		No. B/FB		LI		**No.S./B**		**U. R**	
	Y1	Y2	Y1	Y2	Y1	Y2	Y1	Y2	**Y1**	**Y2**	**Y1**	**Y2**
G. 86 × Suvin	11.69 **	32.57 **	9.81**	29.45 **	17.09 *	-2.66 ns	5.79 *	5.22 *	-0.28 ns	5.43 ns	1.88 **	1.57 **
G. 86 x (G.89 × G. 86)	-12.05 **	2.02 ns	-17.95**	-4.75 ns	5.74 ns	1.53 ns	-11.95 **	-17.33 **	6.53 ns	9.24 **	0.90 ns	0.55 *
G. 86 × G. 94	-41.98 **	-24.30 **	-46.78**	-32.12 **	-12.09 ns	-23.18 **	-21.02 **	-27.53 **	-2.76 ns	15.16 **	2.95 **	2.44 **
G. 86 × G. 96	-7.72 *	-6.80 ns	-10.53**	-9.60 ns	-13.05 ns	-19.85 **	8.97 **	3.27 ns	-15.54 *	4.58 ns	0.74 ns	0.48 *
G. 86 × Pima S6	-23.97 **	-31.36 **	-28.96**	-36.25 **	-17.10 *	-12.22 **	-7.92 **	-14.94 **	-13.75 *	10.23 **	1.54 **	0.35 ns
G. 86 × C.B.58	-2.91 ns	-29.92 **	-6.44 ns	-32.17 **	-18.94 **	-15.00 **	-3.33 ns	-9.34 **	-17.03 *	10.97 **	0.45 ns	2.26 **
G. 86 × TNB	-13.65 **	-20.59 **	-3.97 ns	-14.06 **	-17.03 **	-18.32 **	12.95 **	7.00 **	-3.82 ns	13.29 **	-0.00 ns	-0.27 ns
G. 86 × G.70	-16.82 **	-36.66 **	-16.64**	-37.76 **	-34.80 **	-29.37 **	4.35 ns	9.18 **	-12.03 ns	-5.25 ns	2.04 **	0.35 ns
Kar. x Suvin	-9.86 **	-14.18 *	-15.84**	-20.45 **	5.93 ns	23.39 **	0.34 ns	-12.54 **	-12.83 ns	3.84 ns	1.09 ns	0.16 ns
Kar. x (G.89 × G. 86)	-32.29 **	-36.73 **	-34.81**	-38.52 **	-29.81 **	-24.01 **	-15.39 **	-15.98 **	12.80 ns	11.57 **	0.89 ns	0.75 **
Kar. x G. 94	-37.75 **	-38.24 **	-41.12**	-41.97 **	-23.12 **	-31.60 **	-18.30 **	-18.89 **	-3.18 ns	4.93 ns	0.91 ns	0.51 *
Kar. x G. 96	-27.00 **	-40.03 **	-39.62**	-45.16 **	-11.34 ns	15.23 **	-20.57 **	-22.10 **	-11.72 ns	-7.05 *	1.80 **	2.23 **
Kar. x Pima S6	77.32 **	76.96 **	83.83**	85.71 **	51.25 **	69.10 **	24.03 **	37.49 **	1.67 ns	0.56 ns	1.21 *	-0.02 ns
Kar. x C.B.58	80.74 **	66.84 **	86.04**	77.74 **	41.23 **	82.29 **	15.26 **	15.24 **	0.38 ns	-4.42 ns	0.92 ns	0.42 ns
Kar. x TNB	-1.40 ns	14.15 *	1.5 ns	15.30 *	16.38 *	-0.68 ns	-4.81 *	13.13 **	5.28 ns	-1.66 ns	-0.40 ns	-0.10 ns
Kar. x G.70	17.08 **	1.69 ns	26.32**	7.57 ns	-11.61 ns	36.94 **	17.12 **	22.78 **	-5.97 ns	-21.95 **	-0.84 ns	0.71 **
G.93 × Suvin	11.32 **	24.41 **	12.75**	23.66 **	30.15 **	18.98 **	5.97 *	-0.10 ns	8.24 ns	0.25 ns	-0.14 ns	-0.04 ns
G.93 x (G.89 × G. 86)	-1.59 ns	23.19 **	-2.36 ns	22.54 **	18.78 **	12.05 **	0.79 ns	0.46 ns	18.70 *	-9.35 **	-0.39 ns	0.64 *
G.93 × G. 94	22.01 **	9.59 ns	20.1**	7.71 ns	1.09 ns	19.98 **	-4.24 ns	-11.46 **	25.77 **	16.06 **	0.52 ns	2.53 **
G.93 × G. 96	6.52 ns	2.72 ns	1.05 ns	-2.75 ns	-9.71 ns	-9.99 *	-5.15 *	-7.49 **	24.06 **	14.19 **	-1.57 **	-0.15 ns
G.93 × Pima S6	68.29 **	64.27 **	60.76**	62.54 **	19.81 **	66.94 **	15.26 **	4.66 *	14.60 ns	-3.28 ns	0.73 ns	2.72 **
G.93 × C.B.58	11.89 **	14.12 *	6.94 ns	13.54 *	-15.13 *	12.04 *	-1.83 ns	-5.27 *	24.75 **	2.82 ns	0.48 ns	-0.02 ns
G.93 × TNB	28.50 **	19.38 **	19.83**	13.88 *	17.07 *	42.02 **	-8.85 **	-13.26 **	0.23 ns	-6.46 *	0.42 ns	0.73 **
G.93 × G.70	-7.32 ns	2.64 ns	-3.9 ns	5.64 ns	8.89 ns	25.82 **	8.86 **	12.40 **	-11.75 ns	-25.24 **	0.29 ns	0.11 ns
BBB x Suvin	35.42 **	76.23 **	37.16**	74.80 **	16.26 *	20.00 **	8.06 **	2.83 ns	23.50 **	13.15 **	-0.61 ns	-1.21 **
BBB x (G.89 × G. 86)	-16.71 **	25.08 **	-18.28**	25.71 **	23.17 **	3.31 ns	-6.49 **	-14.85 **	12.12 ns	1.16 ns	-0.86 ns	-1.62 **
BBB x G. 94	29.97 **	68.24 **	31.38**	61.16 **	35.28 **	9.48 *	-8.60 **	-5.16 *	32.52 **	21.72 **	-1.40 *	-1.18 **
BBB x G. 96	-5.05 ns	2.83 ns	-1.03 ns	1.03 ns	-10.61 ns	-1.79 ns	3.21 ns	-2.03 ns	6.14 ns	8.47 **	-0.47 ns	-1.86 **
BBB x Pima S6	82.63 **	79.43 **	72.92**	84.62 **	51.97 **	56.32 **	10.33 **	33.65 **	13.15 ns	-0.66 ns	0.63 ns	-1.46 **
BBB x C.B.58	34.46 **	59.55 **	27.11**	52.31 **	30.55 **	18.14 **	-1.42 ns	3.82 ns	17.30 *	9.03 **	1.19 *	-0.31 ns
BBB x TNB	-14.95 **	-26.07 **	-17.13**	-30.72 **	-34.79 **	-0.29 ns	-15.14 **	-9.92 **	15.75 *	17.49 **	-0.63 ns	0.86 **
BBB x G.70	59.00 **	-2.13 ns	60.09**	-0.89 ns	-23.27 **	35.43 **	9.59 **	14.34 **	12.48 ns	12.37 **	-0.11 ns	-0.85 **

Cross	**F.F. N**		**No.F/B**		P.H		F.F		F.S		UHM	
	**Y1**	**Y2**	**Y1**	**Y2**	Y1	Y2	Y1	Y2	Y1	Y2	Y1	Y2
G. 86 × Suvin	9.36 **	10.30 **	3.43*	5.30 **	21.39 **	30.15 **	0.12 ns	9.44 **	2.36**	-5.79 **	-0.95 ns	4.52 *
G. 86 x (G.89 × G. 86)	4.14 ns	-8.65 **	-10.1**	-3.57 **	4.27 ns	0.87 ns	-16.83 **	-8.48 **	-4.27**	-13.12 **	4.37 **	7.63 **
G. 86 × G. 94	16.51 **	10.11 **	-17.35**	-0.40 ns	7.09 **	14.65 **	-23.14 **	-14.55 **	10.07**	5.17 **	6.96 **	12.25 **
G. 86 × G. 96	-10.91 **	-1.87 ns	6.53**	12.98 **	1.98 ns	0.32 ns	-5.26 **	-11.10 **	-8.95**	-15.96 **	-2.77 *	-0.49 ns
G. 86 × Pima S6	2.71 ns	-8.09 **	-12.83**	0.70 ns	-0.83 ns	0.74 ns	-0.36 ns	7.19 **	3.5**	-7.98 **	0.91 ns	6.55 **
G. 86 × C.B.58	-10.86 **	-8.89 **	9.61**	8.56 **	-7.77 **	-3.29 ns	-3.36 **	1.57 ns	5.33**	-6.58 **	1.23 ns	3.96 ns
G. 86 × TNB	-9.44 **	-26.21 **	-3.68*	-4.66 **	-9.36 **	1.93 ns	-5.82 **	-0.83 ns	9.04**	2.83 **	-1.43 ns	3.55 ns
G. 86 × G.70	-0.02 ns	-9.02 **	17.16**	5.80 **	-4.66 *	-21.68 **	-8.07 **	-16.45 **	0.97 ns	-7.92 **	4.03 **	6.95 **
Kar. x Suvin	-7.65 **	-11.33 **	-24.35**	-13.28 **	-9.88 **	5.81 *	-5.77 **	2.57 **	-3.03**	-8.41 **	-3.66 **	1.42 ns
Kar. x (G.89 × G. 86)	-8.20 **	-7.69 **	-8.34**	-7.68 **	6.02 *	-1.40 ns	1.62 ns	2.99 **	6.04**	1.84 **	-2.27 ns	-2.02 ns
Kar. x G. 94	-8.72 **	-11.54 **	0.42 ns	-5.13 **	-18.45 **	-13.54 **	0.69 ns	-1.46 ns	-0.76 ns	-0.12 ns	-3.70 **	0.59 ns
Kar. x G. 96	8.95 **	4.34 ns	-18.32**	-11.39 **	-16.37 **	-20.02 **	-18.41 **	-11.47 **	5.09**	5.38 **	5.31 **	6.60 **
Kar. x Pima S6	2.09 ns	0.12 ns	-5.84**	-2.82 **	2.26 ns	6.42 *	3.62 **	-8.95 **	7.5**	-1.61 *	-0.50 ns	2.41 ns
Kar. x C.B.58	-11.52 **	-17.76 **	-3.42 ns	8.56 **	8.60 **	4.48 ns	-1.98 *	8.66 **	3.99**	2.76 **	-2.54 ns	3.11 ns
Kar. x TNB	-7.91 **	-9.89 **	-6.04**	-0.92 ns	-16.96 **	-8.34 **	-7.10 **	-5.38 **	7.2**	2.99 **	-1.18 ns	3.32 ns
Kar. x G.70	-11.32 **	-16.08 **	-3.31 ns	10.51 **	0.58 ns	-4.61 *	-0.16 ns	-2.54 **	1.11 ns	-3.74 **	-1.29 ns	-4.02 *
G.93 × Suvin	-17.78 **	-4.46 ns	-3.43*	2.40 **	7.01 **	8.43 **	1.41 ns	20.43 **	3.28**	-7.03 **	0.25 ns	0.10 ns
G.93 x (G.89 × G. 86)	-8.62 **	-7.80 **	-3.62*	6.54 **	13.82 **	23.53 **	1.74 ns	10.24 **	7.78**	-9.20 **	1.28 ns	-2.85 ns
G.93 × G. 94	0.87 ns	5.16 ns	-2.44 ns	8.38 **	-4.11 *	-0.33 ns	-1.46 ns	9.54 **	5.32**	-5.99 **	-0.55 ns	1.36 ns
G.93 × G. 96	-2.56 ns	10.18 **	9.63**	10.82 **	-4.03 ns	-9.24 **	-8.03 **	-10.80 **	-2.23**	-2.22 **	-1.65 ns	-1.58 ns
G.93 × Pima S6	-6.88 *	-13.04 **	-4.55**	1.39 *	-17.42 **	-4.64 ns	-11.26 **	8.24 **	10.87**	-4.84 **	2.03 ns	1.97 ns
G.93 × C.B.58	-13.65 **	-4.04 ns	-2.25 ns	8.22 **	-5.05 *	-14.82 **	-9.29 **	12.70 **	-0.53 ns	-3.82 **	0.96 ns	3.77 ns
G.93 × TNB	-11.82 **	3.91 ns	3.38*	5.70 **	-19.92 **	-8.61 **	-4.96 **	2.21 *	4.01**	0.03 ns	2.99 *	6.76 **
G.93 × G.70	-1.38 ns	-16.12 **	-7.16**	-0.03 ns	-5.91 **	-11.61 **	-11.51 **	-3.90 **	1.2*	-3.90 **	1.82 ns	6.12 **
BBB x Suvin	-21.56 **	-2.38 ns	-1.99 ns	11.00 **	-18.95 **	9.96 **	5.05 **	13.13 **	-5.78**	-9.46 **	-3.58 **	-2.25 ns
BBB x (G.89 × G. 86)	-21.51 **	-2.84 ns	-8.18**	-2.62 **	-5.42 *	7.04 **	8.31 **	13.02 **	-5.6**	-6.49 **	-3.45 **	-1.25 ns
BBB x G. 94	-3.63 ns	19.73 **	4.79**	-0.57 ns	-25.95 **	-14.88 **	6.42 **	-2.81 **	-3.94**	2.85 **	-1.70 ns	-3.74 ns
BBB x G. 96	-4.64 ns	16.15 **	-5.2**	3.07 **	-10.67 **	-2.46 ns	-1.19 ns	2.45 **	-7.55**	-2.83 **	-2.49 ns	1.21 ns
BBB x Pima S6	-7.55 **	4.75 ns	-1.49 ns	-4.69 **	-13.30 **	0.91 ns	6.45 **	16.62 **	0.4 ns	-6.72 **	-0.20 ns	3.53 ns
BBB x C.B.58	-6.19 *	-2.44 ns	-5.08**	-0.57 ns	3.33 ns	7.70 **	8.73 **	11.46 **	4.53**	-2.18 **	-0.10 ns	-1.34 ns
BBB x TNB	-21.89 **	-14.05 **	-20.35**	7.15 **	-24.03 **	-14.42 **	-0.42 ns	-2.38 **	-7.01**	-1.04 ns	-3.20 *	-3.13 ns
BBB x G.70	0.50 ns	-1.76 ns	-8.7**	3.46 **	-14.98 **	-15.60 **	0.21 ns	-3.67 **	7.96**	0.71 ns	-3.80 **	-1.21 ns

^*^and **: significant at 5% and 1% levels of probability, plant height; FF, fiber fineness; FS, fiber strength; UHM, upper half means

### Correlation between studied traits

**Fig. 3:**
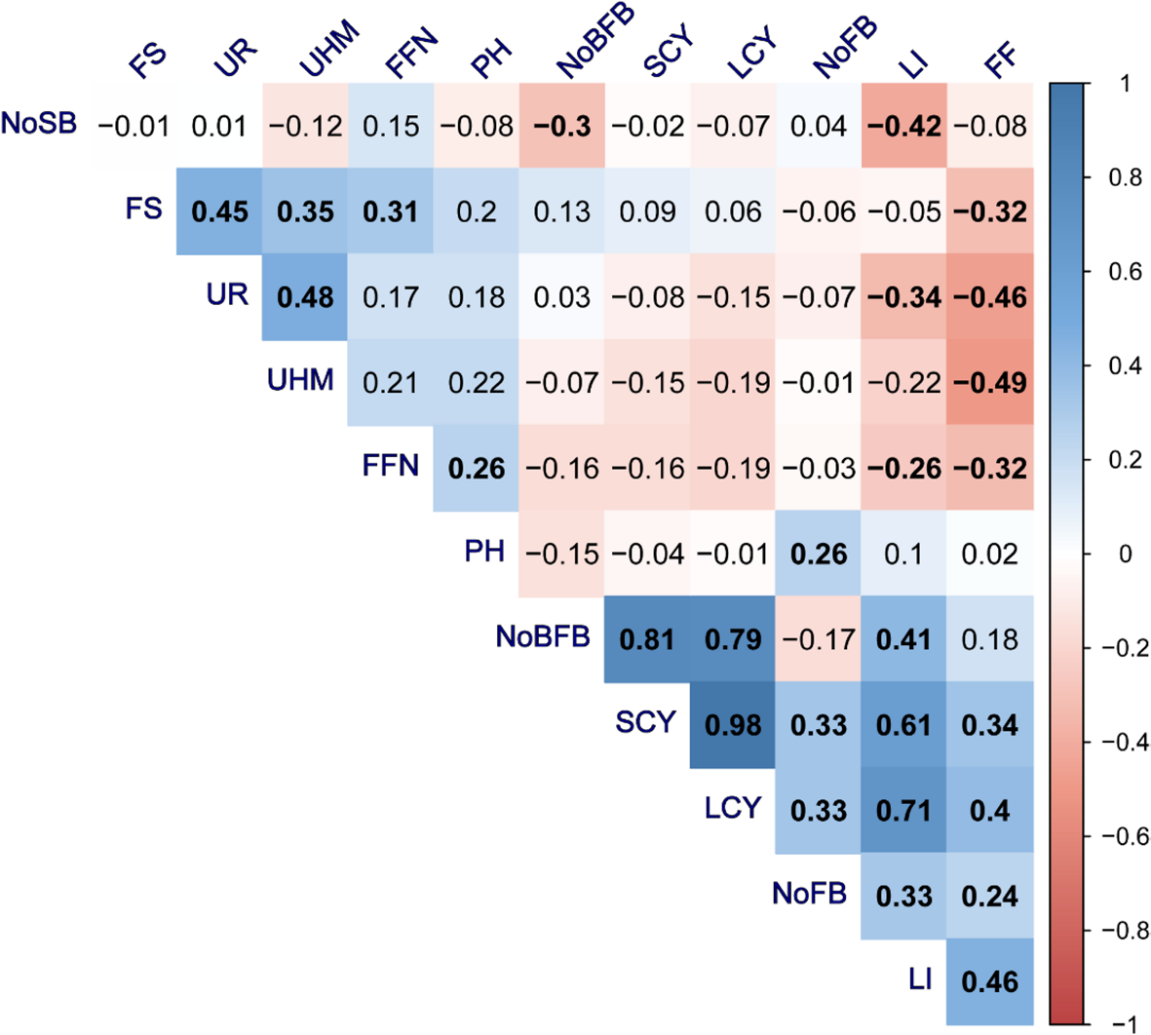
Pearson's genotypic correlation coefficients between the traits. LCY. t/ha., lint cotton yield; SCY. t/ha, seed cotton yield; PH, plant height; No.B/P, number of bolls per plant; No. FB/P, Number of fruiting branches per plant; No.S/B, Number of seeds per boll; LI, lint index; FF, fiber fineness; FS, fiber strength; UR%, uniformity ratio; UHM, upper half means; FFN/P, First Fruiting Node. Blue color indicates positive correlation, while red color indicates negative correlation. Only bold correlation coefficients inside each cell indicate statistically significant correlations (*p* < 0.05)

Correlation analysis among all 12 studied traits (Fig. 3**)** showed that significant positive correlations were exhibited amongst trait pairs, the correlation between No. FB/P and SCY.K/Fad (0.81), SCY. t/ha. and LCY. t/ha. (0.98), No.FB/P and LCY. t/ha (0.79), LCY. t/ha. and L.I. (0.71), S.C.Y. t/ha and LI (0.61) were greatest, while the least correlation was observed between SCY.t/ha, LCY. t/ha and No. FB/P (0.33). Also, correlations among the fiber traits were also significant positive, except for the correlation of FF with each of FS (-0.32), UR% (-0.46), and UHM (-0.49) whereas other pairs of traits showed non-significant correlations, including No. S/B with all traits, FS with PH, SCY. t/ha, LCY. t/ha, LI, No. FB./P, and No. B/FB (Fig. 3).

### Path analysis

**Fig. 4:**
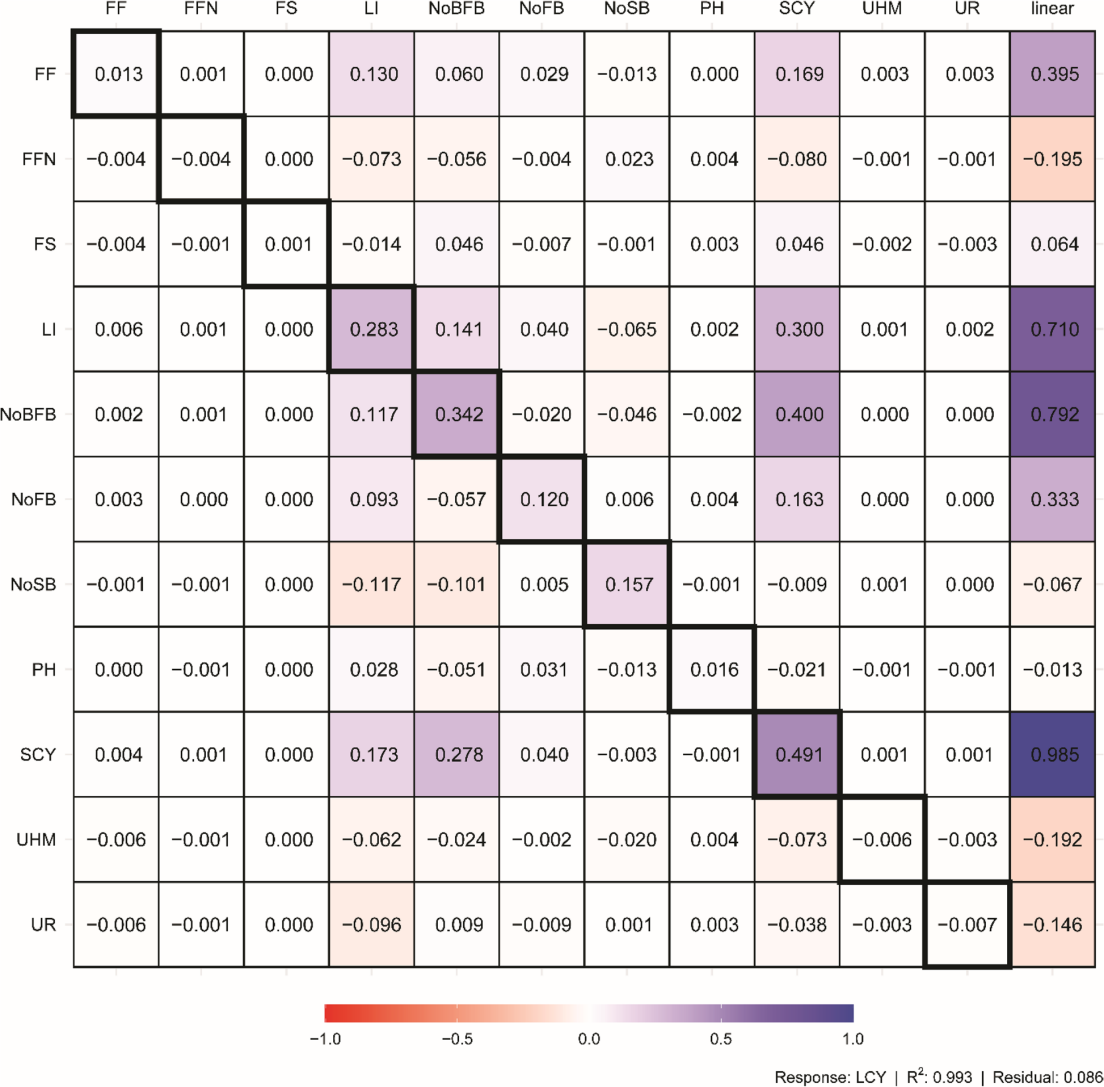
Path diagram showing the direct effect of the 12 explanatory variables on seed cotton yield examined for parents, testers and F1 crosses that evaluated over two seasons of 2016 and 2017. Bidirectional arrows show correlation between the variables, and unidirectional arrows indicate a direct effect on the direction of the arrow, blue and red arrows represent positive and negative effects. Solid arrows indicate *P* ≤ 0.05 and dashed arrows indicate *P* ≥ 0.05. LCY, t/ha. lint cotton yield; SCY. t/ha, seed cotton yield; PH, plant height; No.B/P, number of bolls per plant; No.FB/P, Number of fruiting branches per plant; No.S/B, Number of seeds per boll; LI, lint index; F.F., fiber fineness; FS, fiber strength; UR%, uniformity ratio;UHM, upper half means; FFN/P, First Fruiting Node

The result of direct and indirect correlation coefficients regressed with seed cotton yield was presented in Fig. 4. The path coefficient analysis of indirect and direct effects of the associated traits with lint cotton yield revealed that SCY. t/ha (*r* = 0.49) had the highest indirect contribution to seed cotton yield, followed by NO. B/FB (*r* = 0.40), and LI (*r* = 0.30) indicating the importance of these traits to LCY. t/ha. This needs to be carefully considered simultaneously when selecting for yield improvement in cotton.

## Discussion

The potential of genetic material to transfer the desired features to the offspring plays a key role in the breeding program's ability to enhance crops [28, 29]. Critical selection should be performed to evaluate the inherent potential of the genetic material employed in breeding programs. In this study, eight different cotton genotypes “female parental lines” and four high-yielding testers were crossed to produce 32 hybrids through “line x tester” analysis approach. Before proceeding crossing among these female parental genotypes and testers, cluster analysis was conducted to depict the relationship among the lines and testers used to construct heterotic pools relative to the 12 yield and fiber quality traits evaluated in this study. The findings of cluster analysis revealed wide phenotypic diversity between the female paternal genotypes and testers which, consequently, encouraged to cross among nine parents in this study, leading to the development of super heterotic hybrid cottons. In other words, using cluster analysis in this study confirmed the feasibility of selected varieties to be further manipulated in the cotton breeding program for crossing. Similar results were reported in previous investigation [28, 30, 31].

The four testers were clearly divided into two distinct clusters, which may be due to the variation among those male parents in GCA for most studied traits. Also, the two imported female parent genotypes Giza 70 and Giza 93 were clustered together, showing close relationship and similar performance. On the other side, the parental genotypes Giza 96, Giza 94, BBB, and Giza 89 X Giza 89 were grouped together with narrow genetic base which could be attributed to their performance as poor general combiners for most studied yield and fiber quality traits. Several investigations exploited the phenotypic performance to classify cotton genotypes into different clusters as genotypes from the distant clusters could be favorably utilized in crossing program to obtain a transgressive segregant in early generations [4, 8, 14, 32–37].

The highly significant differences between studied cotton genotypes revealed by ANOVA in the current study for both agronomic and fiber quality traits indicated the existence of genetic diversity among those genotypes. These findings demonstrated the magnitude of utilizing the line x testers method in such cases. For all examined traits, the variance attributable to parents vs. crosses was also statistically significant, indicating the role of hybrid vigor existing for these traits, whereas the line x tester was highly significant, demonstrating the significance of both additive and nonadditive variance. Similar findings were reported in previous investigation on barbadense cotton [1, 38–40]. Collectively, those above findings justified the need to estimate the effects of combining ability between parents (lines and testers) and crosses, respectively.

Twelve yield, yield component, and fiber quality parameters were assessed on the resultant crosses and the twelve parental genotypes. For heterosis breeding to be used effectively, the genetic variance between female parental genotypes and tester lines must be evaluated. Our findings showed that genetic variations among the 44 genotypes were extremely significant (*P* < 0.01) for all characteristics, demonstrating the existence of considerable genetic variation among all genotypes; thus, additional subsequent research was carried out to evaluate the combining ability [31, 41–49]. The bulk of the examined variables also showed extremely significant variations between female parental genotypes, testers, and their interaction. The combining ability of genotypes is practically examined in order to find genotypes with a significant genetic ability for generating cross combinations with desired characteristics and to investigate the gene activity implicated in trait expression [5]. In this context, as documented in previous research on different species, the Line × Tester analysis technique offers a more accurate estimation and valuable prediction of the significant quantitative features, making it a well-established biometrical genetics-based strategy [12, 15, 28, 33, 41].

In general, the fiber length and strength are typically the most important amid all other fiber quality characteristics, such as fiber homogeneity and micronaire [50]. All the 32 cotton crosses shown that fiber length more than 31 mm and less than 34 mm this means that these crosses belonging to long staple category except two crosses Karshenky x Giza 94 and Karshenky x Giza 96 had 35.40- and 35.28-mm fiber length coupled with 10.72 and 10.30 for fiber strength and 4.08 and 3.95 for micronaire reading. Two cotton crosses Karshenky x Pima S6 and Karshenky x C.B.58 had weak values for fiber strength 9.84 and 9.91, respectively. While all the 32 F_1_ cotton crosses were recorded good values for micronaire reading, fiber strength and fiber length. For the most studied traits the performance mean values for both parent and 32 F_1_ hybrids were differed from year to another. Fiber strength appears to improve dramatically due to the variably positioned cellulose microfibrils in the layer. The cotton fiber's secondary wall thereby thickens to 3–6 m [51]. In some *G. hirsutum* cultivars, the thickness of trichome protrusion and fiber initiation are both highly correlated with the lint index and lint percentage [52]. Following initiation, the fiber begins to elongate between 2 to 20 DPA (Days Post Anthesis). As cellulose is synthesized by cellulose synthase between 10 to 16 DPA, which is essential for fiber elongation. The amount of cellulose synthesized, the twisting of fibrils, the orientation of fibers, and the individual length of the cellulose chain all have a direct relationship with fiber elongation and thickness [53, 54]. Remarkably, the pectin biosynthesis genes [55], GhVINI gene [56], expansins [57], aquaporin proteins [58], and their corresponding genes are all activated during the formation of cotton fibers [59].

The two genetic measures, GCA and SCA, are used to measure combining ability and can be influenced by the additive genetic effects and non-allelic interactions of the parents, respectively [5, 35]. In this study, distinct genotypes of both female parental lines and testers showed positive and negative GCA effects, indicating that they were either prospective good combiners or bad combiners in terms of particular studied traits. Given that these genotypes have good general combining ability, female parental genotypes with a great potential to transmit on desirable traits to their cross progeny could be used as a substantial material to improve the qualities of interest [11, 42]. The significant GCA effects reported in the current investigation were consistent with those found in prior studies [31, 60–62]. Positive and significant GCA effects observed in the current study for female parental genotypes and testers are significantly vital. This is because crossings between such promising combiners lead to favorable hybrid combinations in the resulting segregating populations, thus improving certain selections for targeted traits. The high GCA effect could be mainly due to additive gene effects or additive x additive gene interaction effects [44, 63–66]. GCA is therefore advantageous for programs involving selection and hybridization [67, 68]. The production of new synthetic varieties might make use of the parents who showed strong GCA impacts in the desirable direction for certain features [69]. According to Munir et al. [70], traits that are governed by additive genes have a high limited sense of heritability, however such traits may be enhanced by straightforward selection techniques in early segregating generations. The highly significant GCA of LCY.K/Fad female parental lines and testers found in the current study further demonstrate the critical role of the additive nature of gene effects in such trait. It is worth noting that in the current investigation, good combiners parents for SCY.K/Fad were also demonstrated to be good combiners for most of its derived components [71].

Moreover, the results of the current investigation showed that specific combining ability is critical for all yield and fiber quality traits, suggesting that non-additive genetic effects as dominance or epistatic of these traits. These findings clarified the promising role of G.93 × G.70 in terms of LCY.K/Fad, SCY.K/Fad, No. B/P, and No.S/B, while the F1 hybrids Giza 86 × Giza70 and BBB x Pima S6 for FS, FF, UHM, and UR. Based on their performance and significant specific combining potential, these prospective hybrids could be chosen for subsequent recombination breeding projects. Nonetheless, not all F1 hybrid combinations exhibited positive SCA values for all the investigated traits at the same time, indicating that unique hybrid combinations with high significant SCA for numerous variables had both parents with a decent GCA. [11, 35, 43, 72]. In the textile business, fiber quality characteristics are crucial for spinning technology since they affect fiber processing and coloring uniformity [73]. Both yield and fiber quality are intricate quantitative features. While the fiber quality trait is primarily regulated by the gene additive effect and is less affected by epigenetics, the fiber yield trait is controlled by several genes and is strongly influenced by the environment [59, 74]. It was reported that a significant insight is anticipated into the genetic basis of fiber-related properties including fiber strength and fiber length on chromosome 7. Besides, Chromosomes 3, 7, 9, 11, and 12 together with sub genomes A and D were shown to play a substantial role in fiber quality [59]. The fiber quality is affected by specific enzymes such as laccase enzyme that is essential for lignification, plant color, and fiber elongation [75]. The laccase enzyme is variably expressed during various phases of fiber formation, especially at 25 DPA [75].

Interestingly, GCA and SCA variances indicate the amplitude of gene action, which will further aid in developing appropriate breeding strategies for future breeding programs [76]. For some traits, including LCY. t/ha, SCY. t/ha, LI, No. B/P, UHM, and UR%, variances owing to GCA effects were lower than SCA, revealing that non-additive gene action (dominant or epistatic) is important in controlling these traits. For FFN, PH, No.FB/P, No.S/B, FS, and FF, however, GCA variances were greater than SCA variances which emphasize the role of additive genes in comparison to the non-additive genes regulating these traits. These results were consistent with those previously[11, 12, 32, 60, 77].

Correlation analysis was performed to explore dependencies between several variables. As a result, determining the relationships between yield, yield components, and fiber characteristics can aid in the selection of the best breeding technique [78]. Estimation of phenotypic correlations between investigated traits showed that SCY. t/hawas significantly and highly positively correlated with each of No.B./P. and LCY. t/ha, showing that selecting these two traits in a yield improvement program increases lint yield. A similar correlation pattern has been reported in previous studies [79–84]. FFN showed a negative correlation with FF [7, 16, 20, 24, 80]. Similarly, the significant and positive correlation between No. B/FB, and SCY. t/hais advantageous as such increase in boll will in turn lead to an increase the number of seeds per boll, resulting in increasing surface area, which will enhance the maximum lint percentage. The current study, like prior studies, revealed negative correlations among yield related and fiber quality traits. Correlation between SCY. t/ha, and LCY. t/hawith fiber quality traits showed non-significant association [3, 9, 83]. Such negative association revealed between FS, and FF could be due to the repulsive linkage [61, 72, 80, 85–89]. In contrast, the positive and significant associations between FS and UR% observed in our study further indicates that any of the traits could be used for direct selection for genotypes with good fiber quality and technological properties which was in line with previously reported findings [40, 82]**.**

Although correlation coefficients are important, they may be deceptive regarding the link between two traits and are not always a reliable indicator of cause and effect. As a result, the strength and effectiveness of the correlation coefficient between two traits may be explained by the impact of a third trait or set of traits, which does not precisely indicate the relative importance of the direct and indirect impacts of the traits under study [33, 43]. Such issue has led the current study to use path analysis. Strikingly, the largest direct effect of SCY.t/ha on the dependent variable LCY. t/ha in this study implied that SCY.t/ha could be used as marker to improve LCY.t/ha through direct selection process. These findings were in line with recent investigations[2, 33, 35, 85]. Furthermore, No.B/P showed the highest positive indirect contribution to lint cotton yield through SCY.t/ha followed by LI through No.S/B, No.B/FB through SI which pinpoints the importance of these traits due to their indirect important role on improvement of LCY. t/ha. Such findings suggest that while selecting for yield improvement strategies in cotton, careful and simultaneous attention should be taken, and that selection for LCY and t/ha should also be based on such marker traits.

To better exploit parent heterosis for the aforementioned collection of characteristics, these crossings must be taken into account for hybrid development programs [90]. The existence of diversity in the parents' performance in comparison to hybrid development programs may be linked to variations in the plant genomic constitution. The existence of non-additive gene action for the examined traits demonstrated that the available genetic material might be a viable choice for the creation of hybrids [91]. Additionally, developing high-yielding cotton genotypes may be possible by inter-specific crossing of elite, landraces, and wild relatives’ cotton [92–94].

GCA and SCA variations were significant, demonstrating the significance of both additive and non-additive gene activities in regulating the investigated characteristics. The GCA has been linked to the additive gene activities as a guide to the utility of a genotype in hybrid combinations. The SCA, on the other hand, is useful for creating possible hybrids with greater performance and a reasonable level of stability; variations in the SCA have been related to non-additive (dominance or epistasis) gene activity [39, 95]. GCA is therefore advantageous for programs involving selection and hybridization, whereas SCA is advantageous for the creation of hybrids and heterotic effects [67, 68]. The fact that SCA variation was higher than GCA variation indicates that non-additive gene activity played a bigger role in determining the examined features [39]. The higher values of GCA, SCA, and heterosis suggest the greater likelihood of choosing the future genotype for certain features that might be used to increase cotton yield and fiber quality [25]. Consequently, these genotypes were viewed as being prospective for enhancing cotton qualities in breeding efforts.

### Future perspective

The cotton hybrids discovered in this work can be used as a source of high-yielding germplasm. By using marker-assisted parental selection to find the marker associated with the genes that the best values of targeted traits may be introduced. Genome-assisted backcrossing can then be used to effectively transfer those genes into other germplasms. Utilizing genome editing methods like CRISPR-Cas9, which don't produce GMOs and are generally recognized as non-transgenic, it is possible to transfer the gene [94]. Modern analytical techniques like machine learning, genomic prediction, and multi trait gene editing can precisely speed up the breeding efforts to boost crop productivity [94]. Also, the whole-genome sequencing could offer full knowledge on fiber and fiber-related properties [59]. Studies on the cotton genome, DNA markers, and transcriptome demonstrate the important function played by many chromosomes that include genes related to the fiber quality. Furthermore, the expression profiles and sequence of candidate genes are demonstrated by RT-qPCR and molecular cloning [59]. Global fiber and food security in the future will depend on the proper use of wild germplasm, which will require big data analytics created through transdisciplinary techniques employing open-source data and long-term investments [96]. To increase cotton productivity in Egypt, it is primarily necessary to reinvigorate cotton hybrid development.

## Conclusion

The variances of all source variation determined by line-x tester analysis revealed that the majority of evaluated features differed significantly. The interaction lines × testers made a significant contribution to the expression variances for most studied traits. The lines Suvin, and Pima S6 as well as the tester BBB remarkably increased cotton yield and most investigated traits compared to the other lines and testers. Finally, the three hybrids above are considered potential promising genotypes to be best exploited and utilized in future cotton breeding efforts to generate hybrid cotton and increase yield, its constituent parts, and fiber quality attributes.

## Data Availability

All the data are available in the manuscript and with Correspondence authors.

## References

[1] Yehia W, El-Hashash EF (2022). Estimates of genetic parameters for cotton yield, its components, and fiber quality traits based on line x tester mating design and principal component analysis. Egyptian Journal of Agricultural Research.

[2] El-Aty A, Hamoud H, Omar A, Turkey HS. Estimation of genetic variability in some cotton crosses (Gossypium Barbadense l.) Under water stress. J Plant Prod. 2012;3(6):1017–26.

[3] Fasoula DA, Fasoula VA (1997). Gene action and plant breeding. Plant Breeding Reviews.

[4] Geng X, Sun G, Qu Y, Sarfraz Z, Jia Y, He S, Pan Z, Sun J, Iqbal MS, Wang Q (2020). Genome-wide dis The Plant Journalsection of hybridization for fiber quality-and yield-related traits in upland cotton. Plant J.

[5] Sprague GF, Tatum L. General vs. specific combining ability in single crosses of corn. J Am Soc Agronomy. 1942;14(10):923–32.

[6] Narayanan A, Wang D. Ideal ratio mask estimation using deep neural networks for robust speech recognition. In: 2013 IEEE International Conference on Acoustics, Speech and Signal Processing: 2013. IEEE International Conference on Acoustics, Speech and Signal Processing: May 26, 7092–7096. 10.1109/ICASSP.2013.6639038.

[7] Basbag S, Ekinci R, Gencer O (2007). Combining ability and heterosis for earliness characters in line× tester population of Gossypium hirsutum L. Hereditas.

[8] Geng X, Qu Y, Jia Y, He S, Pan Z, Wang L, Du X (2021). Assessment of heterosis based on parental genetic distance estimated with SSR and SNP markers in upland cotton (Gossypium hirsutum L.). BMC genomics.

[9] Fasahat P, Rajabi A, Rad JM, Derera J. Principles and utilization of combining ability in plant breeding. Biometrics Biostatistics Int J. 2016;4(1):1–24.

[10] Prakash G, Korekar S, Mankare S (2018). Combining ability analysis in Bt cotton (G. hirsutum L.) to harness high yield under contrasting planting densities through heterosis breeding. International Journal of Current Microbiology Applied Sciences.

[11] Han Y-y, Wang K-y, Liu Z-q, Pan S-h, Zhao X-y (2020). Zhang Q, Wang S-f: Research on hybrid crop breeding information management system based on combining ability analysis. Sustainability.

[12] Jain S, Sastry E (2012). Heterosis and combining ability for grain yield and its contributing traits in bread wheat (Triticum aestivum L.). Journal of Agriculture Allied Science.

[13] Akaogu I, Badu-Apraku B, Adetimirin V (2013). VROH-BI I, Oyekunle M, Akinwale R: Genetic diversity assessment of extra-early maturing yellow maize inbreds and hybrid performance in Striga-infested and Striga-free environments. J Agric Sci.

[14] Ali M, Mian M, Rasul M, Miah MA, Alam M, Hossain M (2012). Genetic diversity in local aromatic rice (Oryza sativa L.) genotypes. Bangladesh Journal of Plant Breeding Genetics.

[15] Kempthorne O. An introduction to genetic statistics. xvii, 545, Oxford: wiley; 1957.

[16] Kumar P, Nimbal S, Sangwan RS, Budhlakoti N, Singh V, Mishra DC, Choudhary RR (2021). Identification of novel marker–trait associations for lint yield contributing traits in upland cotton (Gossypium hirsutum L) using SSRs. Front plant sci.

[17] Malik W, Khan AA (2013). Sadia BJAJoCS: In situ characterization of coloured cotton genotypes. Aust J Crop Sci.

[18] Méndez-Natera JR, Rondón A, Hernández J, Merazo-Pinto JFJS (2012). Genetic studies in upland cotton. III. Genetic parameters, correlation and path analysis. Breeding Genetics.

[19] Patel HR, Patel D (2018). Heterotic analysis of GMS based hybrids of seed cotton yield and fiber quality traits in cotton (Gossypium hirsutum, L.). International Journal of Chemical Studies.

[20] Imran M, Saif-ul-Malook SA, Nawaz MA, Ahabaz M, Asif M, Ali Q (2015). Combining ability analysis for yield related traits in sunflower (Helianthus annuus L.). American-Eurasian J Agric Environ Sci.

[21] Mohammed R, Are AK, Bhavanasi R, Munghate RS, Kavi Kishor PB, Sharma HC (2015). Quantitative genetic analysis of agronomic and morphological traits in sorghum. Sorghum bicolor Frontiers in plant science.

[22] Mokadem S, Salem M, Khalifa H, Salem T. COMBINING ABILITY, Heterosis And Heritability For Fiber Quality Properties In Egyptian Cotton Crosses. Egyptian J Plant Breeding. 2020;24 (1). 10.12816/ejpb.2020.199305.

[23] Xiaoquan Z, Xuede W, Dutt Y. Improvement in yield and fibre quality using interspecific hybridization in cotton (Gossypium spp). Indian Council Agricutural Res. 2011:0019–5022.

[24] Jenkins JN, McCarty JC, Wu J, Hayes R, Stelly D (2012). Genetic effects of nine Gossypium barbadense L. chromosome substitution lines in top crosses with five elite Upland cotton G. hirsutum L. cultivars. Euphytica.

[25] Amer EA. Identifying Superior Parents and Hybrids for Yield, Its Components and Fiber Quality in Cotton (Gossypium barbadense L.). Egyptian J Agronomy. 2022;44(1):59–73.

[26] Steel RGD, Torrie JH. Principles and procedures of statistics, a biometrical approach: McGraw-Hill Kogakusha, Ltd.; 1980.

[27] Zafar MM, Jia X, Shakeel A, Sarfraz Z, Manan A, Imran A, Mo H, Ali A, Youlu Y, Razzaq A (2022). Unraveling heat tolerance in upland cotton (Gossypium hirsutum L.) using univariate and multivariate analysis. Front Plant Sci.

[28] Zafar MM, Manan A, Razzaq A, Zulfqar M, Saeed A, Kashif M, Khan AI, Sarfraz Z, Mo H, Iqbal MS (2021). Exploiting Agronomic and Biochemical Traits to Develop Heat Resilient Cotton Cultivars under Climate Change Scenarios. Agronomy.

[29] Razzaq A, Zafar MM, Li P, Qun G, Deng X, Ali A, et al. Transformation and Overexpression of Primary Cell Wall Synthesis-Related Zinc Finger Gene Gh_A07G1537 to Improve Fiber Length in Cotton. Front Plant Sci. 2021:2561. 10.3389/fpls.2021.777794 PMC8604042 34804108

[30] Abdelghany AM, Zhang S, Azam M, Shaibu AS, Feng Y, Qi J, Li J, Li Y, Tian Y, Hong H (2021). Exploring the phenotypic stability of soybean seed compositions using multi-trait stability index approach. Agronomy.

[31] Youssef MA, Yousef AF, Ali MM, Ahmed AI, Lamlom SF, Strobel WR, Kalaji HM (2021). Exogenously applied nitrogenous fertilizers and effective microorganisms improve plant growth of stevia (Stevia rebaudiana Bertoni) and soil fertility. AMB Express.

[32] Akter T, Islam A, Rasul M, Kundu S, Ahmed J (2019). Evaluation of genetic diversity in short duration cotton (Gossypium hirsutum L.). Journal of Cotton Research.

[33] El-Mowafi HF, AlKahtani MD, Abdallah RM, Reda AM, Attia KA, El-Hity MA, El-Dabaawy HE, Husnain LA, Al-Ateeq TK (2021). EL-Esawi MA: Combining ability and gene action for yield characteristics in novel aromatic cytoplasmic male sterile hybrid rice under water-stress conditions. Agriculture.

[34] Vasconcelos WS (2020). Santos RCd, Vasconcelos UA, Cavalcanti JJ, Farias FJ: Estimates of genetic parameters in diallelic populations of cotton subjected to water stress. Revista Brasileira de Engenharia Agrícola e Ambiental.

[35] Elmardy NA, Yousef AF, Lin K, Zhang X, Ali MM, Lamlom SF, Kalaji HM, Kowalczyk K, Xu Y (2021). Photosynthetic performance of rocket (Eruca sativa. Mill.) grown under different regimes of light intensity, quality, and photoperiod. Plos one..

[36] Nasar J, Wang G-Y, Ahmad S, Muhammad I, Zeeshan M, Gitari H, Adnan M, Fahad S, Khalid MHB, Zhou X-B (2022). Nitrogen fertilization coupled with iron foliar application improves the photosynthetic characteristics, photosynthetic nitrogen use efficiency, and the related enzymes of maize crops under different planting patterns. Front Plant Sci.

[37] Hassan MU, Ghareeb RY, Nawaz M, Mahmood A, Shah AN, Abdel-Megeed A, Abdelsalam NR, Hashem M, Alamri S, Thabit MA (2022). Melatonin: a vital pro-tectant for crops against heat stress: mechanisms and prospects. Agronomy.

[38] Kaushik P, Dhaliwal MS (2018). Diallel analysis for morphological and biochemical traits in tomato cultivated under the influence of tomato leaf curl virus. Agronomy.

[39] Yehia W, El-Hashash E. Combining ability effects and heterosis estimates through line x tester analysis for yield, yield components and fiber traits in Egyptian cotton. J Agronomy. 2019;12:10.

[40] Abdelsalam NR, Botros WA, Khaled AE, Ghonema MA, Hussein SG, Ali HM, Elshikh MS (2019). Comparison of uridine diphosphate-glycosyltransferase UGT76G1 genes from some varieties of Stevia rebaudiana Bertoni. Sci Rep.

[41] Karademir E, Karademir Ç, Başal H (2016). Combining Ability and Line x Tester Analysis on Heat Tolerance in Cotton (Gossypium hirsutum L.). Indian Journal Of Natural Sciences.

[42] Gheith E, El-Badry OZ, Kandil EE, Lamlom SF, Abdelsalam NR (2022). Maize (Zea mays L.) productivity and nitrogen use efficiency in response to nitrogen application levels and time. Front Plant Sci.

[43] Lamlom SF, Zhang Y, Su B, Wu H, Zhang X, Fu J, Zhang B (2020). QIU L-j: Map-based cloning of a novel QTL qBN-1 influencing branch number in soybean [Glycine max (L.) Merr.]. The Crop Journal.

[44] Ochar K, Su BH, Zhou MM, Liu ZX, GAO H-w, Lamlom SF, Qiu LJ. Identification of the genetic locus associated with crinkled leaf phenotype in a soybean (Glycine max L.) mutant by BSA-Seq technology. J Integr Agric. 2022;21(12):3524–39.

[45] Samar J, Butt GY, Shah AA, Shah AN, Ali S, Jan BL, Abdelsalam NR, Hussaan M. Phycochemical and biological activities from different extracts of Padina antillarum (Kützing) Piccone. Front Plant Sci. 2022;13. 10.3389/fpls.2022.929368. 10.3389/fpls.2022.929368 PMC9354264 35937357

[46] Tabussam N, Rana RM, Wattoo FM, Khan AI, Amir RM, Javed T, Ahmar S, Dessoky ES, Abdelsalam NR (2022). Single nucleotide polymorphism based assessment of genetic diversity in local and exotic onion genotypes. Mol Biol Rep.

[47] Iqbal Z, Javad S, Naz S, Shah AA, Shah AN, Paray BA, Gulnaz A, Abdelsalam NR. Elicitation of the in vitro Cultures of Selected Varieties of Vigna radiata L. With Zinc Oxide and Copper Oxide Nanoparticles for Enhanced Phytochemicals Production. Front Plant Sci. 2022:13. 10.3389/fpls.2022.908532. 10.3389/fpls.2022.908532 PMC9360770 35958222

[48] Ullah A, Shakeel A, Ahmed HGM-D, Naeem M, Ali M, Shah AN, Wang L, Jaremko M, Abdelsalam NR, Ghareeb RY. Genetic basis and principal component analysis in cotton (Gossypium hirsutum L.) grown under water deficit condition. 2022. 10.3389/fpls.2022.981369. 10.3389/fpls.2022.981369 PMC9583382 36275586

[49] Ali A, Altaf MT, Nadeem MA, Karaköy T, Shah AN, Azeem H, Baloch FS, Baran N, Hussain T, Duangpan S. Recent advancement in OMICS approaches to enhance abiotic stress tolerance in legumes. Front Plant Sci. 2022:13. 10.3389/fpls.2022.952759. 10.3389/fpls.2022.952759 PMC9554552 36247536

[50] Yang X, Wang Y, Zhang G, Wang X, Wu L, Ke H, Liu H, Ma Z (2016). Detection and validation of one stable fiber strength QTL on c9 in tetraploid cotton. Mol Genet Genomics.

[51] Hinchliffe DJ, Meredith WR, Delhom CD, Thibodeaux DP, Fang DD (2011). Elevated growing degree days influence transition stage timing during cotton fiber development resulting in increased fiber-bundle strength. Crop Sci.

[52] Li C, Guo W, Zhang T (2009). Fiber initiation development in Upland cotton (Gossypium hirsutum L.) cultivars varying in lint percentage. Euphytica.

[53] Betancur L, Singh B, Rapp RA, Wendel JF, Marks MD, Roberts AW, Haigler CH (2010). Phylogenetically distinct cellulose synthase genes support secondary wall thickening in Arabidopsis shoot trichomes and cotton fiber. J Integr Plant Biol.

[54] Haigler CH, Betancur L, Stiff MR, Tuttle JR (2012). Cotton fiber: a powerful single-cell model for cell wall and cellulose research. Front Plant Sci.

[55] Pang C-Y, Wang H, Pang Y, Xu C, Jiao Y, Qin Y-M, Western TL, Yu S-X, Zhu Y-X (2010). Comparative proteomics indicates that biosynthesis of pectic precursors is important for cotton fiber and Arabidopsis root hair elongation. Mol Cell Proteomics.

[56] Wang L, Li X-R, Lian H, Ni D-A (2010). He Y-k, Chen X-Y, Ruan Y-L: Evidence that high activity of vacuolar invertase is required for cotton fiber and Arabidopsis root elongation through osmotic dependent and independent pathways, respectively. Plant Physiol.

[57] Li C, Dong Y, Zhao T, Li L, Li C, Yu E, Mei L, Daud MK, He Q, Chen J, et al. Genome-Wide SNP Linkage Mapping and QTL Analysis for Fiber Quality and Yield Traits in the Upland Cotton Recombinant Inbred Lines Population. Front Plant Sci. 2016:7. 10.3389/fpls.2016.01356. 10.3389/fpls.2016.01356 PMC5014859 27660632

[58] Liu D, Tu L, Wang L, Li Y, Zhu L, Zhang X (2008). Characterization and expression of plasma and tonoplast membrane aquaporins in elongating cotton fibers. Plant Cell Rep.

[59] Razzaq A, Zafar MM, Ali A, Hafeez A, Sharif F, Guan X, Deng X, Pengtao L, Shi Y, Haroon M. The pivotal role of major chromosomes of sub-genomes A and D in fiber quality traits of cotton. Front Genet. 2021:12. 10.3389/fgene.2021.642595. 10.3389/fgene.2021.642595 PMC8988190 35401652

[60] Borzan G (2021). GÜVERCİN RŞ: COMBINING ABILITY AND HYBRID POWER IN INTERSPECIFIC (Gossypium hirsutum L. x Gossypium barbadense L.) LINE x TESTER HYBRIDS OF COTTON. Turkish Journal Of Field Crop.

[61] Mangi N, Nazir MF, Wang X, Iqbal MS, Sarfraz Z, Jatoi GH, Mahmood T, Ma Q, Shuli F (2021). Dissecting Source-Sink Relationship of Subtending Leaf for Yield and Fiber Quality Attributes in Upland Cotton (Gossypium hirsutum L.). Plants.

[62] Shaibu AS, Badu-Apraku B, Ayo-Vaughan MA (2021). Enhancing drought tolerance and Striga hermonthica resistance in maize using newly derived inbred lines from the wild maize relative, Zea diploperennis. Agronomy.

[63] Khokhar E, Shakeel A, Maqbool M, Abuzar M, Zareen S, Aamir S, Asadullah M (2018). Studying combining ability and heterosis in different cotton (Gossypium hirsutum L.) genotypes for yield and yield contributing traits. Pakistan Journal of Agricultural Research.

[64] Verma S, Goyal S, Tuteja O (2021). Line x tester mating design analysis with GMS based system for seed cotton yield, its component traits and fibre quality parameters in Asiatic cotton (Gossypium arboreum L.). Electronic Journal of Plant Breeding.

[65] Singh R. Biometrical methods in quantitative genetic analysis. Kalyani Pub. Ludhiana. New Delhi, Revised Ed. 1985:318. https://cir.nii.ac.jp/crid/1572543024702551552.

[66] Zhang X, Yong H, Zhou Z, Zhang C, Lu M, Sun Q, Zhang L, Li M, Zhang D, Weng J (2017). Heterosis and combining ability of seven maize germplasm populations. Euphytica.

[67] Jatoi WA, Memon S. Line x tester analysis for earliness and yield traits in cotton (Gossypium hirsutum L.). J Agric Res. 2016;54(4):615–29.

[68] Panhwar S, Baloch M, Jatoi W, Veesar N, Majeedano M (2008). Combining ability estimates from line x tester mating design in upland cotton. Pak Acad Sci.

[69] Amer E, Hassan S, Abo-Hegazy S (2021). Genetic analysis for yield and fiber quality traits in egyptian cotton (Gossypium barbadense L.). Egyptian Journal of Plant Breeding.

[70] Munir S, Qureshi MK, Shahzad AN, Manzoor H, Shahzad MA, Aslam K (2018). Assessment of gene action and combining ability for fibre and yield contributing trais in interspecific and intraspecific hybrids of cotton. Czech Journal of Genetics and Plant Breeding.

[71] Richika R, Rajeswari S, Premalatha N, Thirukumaran K (2021). Heterosis and combining ability analysis for yield contributing traits and fibre quality traits in interspecific cotton hybrids (Gossypium hirsutum L. x Gossypium barbadense L.). Electronic Journal of Plant Breeding.

[72] Zhao L, Liu S, Abdelsalam NR, Carver BF, Bai G (2021). Characterization of wheat curl mite resistance gene Cmc4 in OK05312. Theoretical Applied Genetics.

[73] Rodgers J, Zumba J, Fortier C (2017). Measurement comparison of cotton fiber micronaire and its components by portable near infrared spectroscopy instruments. Text Res J.

[74] Zhang J, Abdelraheem A, Wu J (2017). Heterosis, combining ability and genetic effect, and relationship with genetic distance based on a diallel of hybrids from five diverse Gossypium barbadense cotton genotypes. Euphytica.

[75] Balasubramanian VK, Rai KM, Thu SW, Hii MM, Mendu V (2016). Genome-wide identification of multifunctional laccase gene family in cotton (Gossypium spp.); expression and biochemical analysis during fiber development. Sci rep.

[76] Kaushik P, Dhaliwal MSJA (2018). Diallel analysis for morphological and biochemical traits in tomato cultivated under the influence of tomato leaf curl virus.

[77] Koebernick JC, Liu S, Constable GA, Stiller WN (2019). Parental selection strategy for improving fibre strength and maintaining lint yield in cotton. Industrial Crops Products.

[78] Shahzad K, Li X, Qi T, Guo L, Tang H, Zhang X, Wang H, Zhang M, Zhang B (2019). QIAO X: Genetic analysis of yield and fiber quality traits in upland cotton (Gossypium hirsutum L.) cultivated in different ecological regions of China. Journal of Cotton Research.

[79] Abbas A, Shah AN, Shah AA, Nadeem MA, Alsaleh A, Javed T, Alotaibi SS, Abdelsalam NR (2022). Genome-Wide Analysis of Invertase Gene Family, and Expression Profiling under Abiotic Stress Conditions in Potato. Biology.

[80] Abdelsalam NR, Ali HM, Salem MZ, Ibrahem EG, Elshikh MS (2018). Genetic and morphological characterization of Mangifera indica L. growing in Egypt. HortScience.

[81] Ahmed HB, Abualnaja KM, Ghareeb RY, Ibrahim AA, Abdelsalam NR, Emam HE (2021). Technical textiles modified with immobilized carbon dots synthesized with infrared assistance. Journal of colloid interface science.

[82] Alam MS, Kong J, Tao R, Ahmed T, Alamin M, Alotaibi SS, Abdelsalam NR, Xu J-H (2022). CRISPR/Cas9 Mediated Knockout of the OsbHLH024 Transcription Factor Improves Salt Stress Resistance in Rice (Oryza sativa L.). Plants.

[83] Fouda MM, Abdelsalam NR, Gohar I, Hanfy AE, Othman SI, Zaitoun AF, Allam AA, Morsy OM, El-Naggar M (2020). Utilization of High throughput microcrystalline cellulose decorated silver nanoparticles as an eco-nematicide on root-knot nematodes. Colloids Surfaces B: Biointerfaces.

[84] Mosa WF, El-Shehawi AM, Mackled MI, Salem MZ, Ghareeb RY, Hafez EE, Behiry SI, Abdelsalam NR (2021). Productivity performance of peach trees, insecticidal and antibacterial bioactivities of leaf extracts as affected by nanofertilizers foliar application. Sci Rep.

[85] Abdelsalam NR, Abdel-Megeed A, Ali HM, Salem MZ, Al-Hayali MF, Elshikh MS (2018). Genotoxicity effects of silver nanoparticles on wheat (Triticum aestivum L.) root tip cells. Ecotoxicology environmental safety.

[86] Choudhary RC, Bairwa H, Kumar U, Javed T, Asad M, Lal K, Mahawer L, Sharma S, Singh P, Hassan MM (2022). Influence of organic manures on soil nutrient content, microbial population, yield and quality parameters of pomegranate (Punica granatum L.) cv. Bhagwa. Plos one.

[87] El-Naggar ME, Abdelsalam NR, Fouda MM, Mackled MI, Al-Jaddadi MA, Ali HM, Siddiqui MH, Kandil EE (2020). Soil application of nano silica on maize yield and its insecticidal activity against some stored insects after the post-harvest. Nanomaterials.

[88] Ghareeb RY, Alfy H, Fahmy AA, Ali HM, Abdelsalam NR (2020). Utilization of Cladophora glomerata extract nanoparticles as eco-nematicide and enhancing the defense responses of tomato plants infected by Meloidogyne javanica. Sci Rep.

[89] Hossain MI, Soliman MM, El-Naggar ME, Sultan MZ, Kechi A, Abdelsalam NR, Abu-Saied M, Chowdhury M (2021). Synthesis and characterization of graphene oxide-ammonium ferric sulfate composite for the removal of dyes from tannery wastewater. Journal of Materials Research Technology.

[90] Shakeel A, Naeem M, İmtiaz A (2017). ALLAH SU, Afzal I, Saeed A, Iqbal M: Genetic mechanism controlling selected within boll yield components and physiological traits of Gossypium hirsutum L. under salinity stress. Turkish Journal of Field Crops.

[91] Zafar MM, Razzaq A, Farooq MA, Rehman A, Firdous H, Shakeel A, Mo H, Ren M, Ashraf M, Youlu Y. Genetic Variation Studies of Ionic and within Boll Yield Components in Cotton (Gossypium Hirsutum L.) Under Salt Stress. J Nat Fibers. 2022;19(8):3063–82.

[92] Burbano-Erazo E, León-Pacheco RI, Cordero-Cordero CC, López-Hernández F, Cortés AJ, Tofiño-Rivera AP (2021). Multi-environment yield components in advanced common bean (Phaseolus vulgaris L.)× tepary bean (P. acutifolius A. Gray) interspecific lines for heat and drought tolerance. Agronomy.

[93] Buitrago-Bitar MA, Cortés AJ, López-Hernández F, Londoño-Caicedo JM, Muñoz-Florez JE, Muñoz LC, Blair MW (2021). Allelic diversity at abiotic stress responsive genes in relationship to ecological drought indices for cultivated tepary bean, Phaseolus acutifolius A. Gray, and its wild relatives. Genes.

[94] Zafar MM, Zhang Y, Farooq MA, Ali A, Firdous H, Haseeb M, Fiaz S, Shakeel A, Razzaq A, Ren M (2022). Biochemical and Associated Agronomic Traits in Gossypium hirsutum L. under High Temperature Stress. Agronomy.

[95] Griffing B (1956). Concept of general and specific combining ability in relation to diallel crossing systems. Aust J Biol Sci.

[96] Cortés AJ, López-Hernández F (2021). Harnessing crop wild diversity for climate change adaptation. Genes.

